# Speech Reception in Young Children with Autism Is Selectively Indexed by a Neural Oscillation Coupling Anomaly

**DOI:** 10.1523/JNEUROSCI.0112-22.2023

**Published:** 2023-10-04

**Authors:** Xiaoyue Wang, Jaime Delgado, Silvia Marchesotti, Nada Kojovic, Holger Franz Sperdin, Tonia A. Rihs, Marie Schaer, Anne-Lise Giraud

**Affiliations:** ^1^Auditory Language Group, Department of Basic Neuroscience, University of Geneva, Geneva, Switzerland, 1202; ^2^Autism Brain & Behavior Lab, Department of Psychiatry, University of Geneva, Geneva, Switzerland, 1202; ^3^Functional Brain Mapping Laboratory, Department of Basic Neuroscience, University of Geneva, Geneva, Switzerland, 1202; ^4^Institut Pasteur, Université Paris Cité, Hearing Institute, Paris, France, 75012

**Keywords:** Autism spectrum disorder (ASD) children, Typically developing (TD) children, speech processing, low-frequency neural oscillations, neural tracking, phase-amplitude coupling (PAC)

## Abstract

Communication difficulties are one of the core criteria in diagnosing autism spectrum disorder (ASD), and are often characterized by speech reception difficulties, whose biological underpinnings are not yet identified. This deficit could denote atypical neuronal ensemble activity, as reflected by neural oscillations. Atypical cross-frequency oscillation coupling, in particular, could disrupt the joint tracking and prediction of dynamic acoustic stimuli, a dual process that is essential for speech comprehension. Whether such oscillatory anomalies already exist in very young children with ASD, and with what specificity they relate to individual language reception capacity is unknown. We collected neural activity data using electroencephalography (EEG) in 64 very young children with and without ASD (mean age 3; 17 females, 47 males) while they were exposed to naturalistic-continuous speech. EEG power of frequency bands typically associated with phrase-level chunking (δ, 1–3 Hz), phonemic encoding (low-γ, 25–35 Hz), and top-down control (β, 12–20 Hz) were markedly reduced in ASD relative to typically developing (TD) children. Speech neural tracking by δ and θ (4–8 Hz) oscillations was also weaker in ASD compared with TD children. After controlling gaze-pattern differences, we found that the classical θ/γ coupling was replaced by an atypical β/γ coupling in children with ASD. This anomaly was the single most specific predictor of individual speech reception difficulties in ASD children. These findings suggest that early interventions (e.g., neurostimulation) targeting the disruption of β/γ coupling and the upregulation of θ/γ coupling could improve speech processing coordination in young children with ASD and help them engage in oral interactions.

**SIGNIFICANCE STATEMENT** Very young children already present marked alterations of neural oscillatory activity in response to natural speech at the time of autism spectrum disorder (ASD) diagnosis. Hierarchical processing of phonemic-range and syllabic-range information (θ/γ coupling) is disrupted in ASD children. Abnormal bottom-up (low-γ) and top-down (low-β) coordination specifically predicts speech reception deficits in very young ASD children, and no other cognitive deficit.

## Introduction

Autism spectrum disorder (ASD) primarily refers to disorders of social interactions; however, individuals with ASD are seldom spared from language difficulties. Many people with ASD have severe language impairment, and even high-functioning ASD individuals with excellent language skills have difficulty understanding speech in noisy environments and/or when exposed to multiple speakers (Beker et al., 2018; Schelinski and von Kriegstein, 2020). Individuals with ASD occasionally report that during childhood, speech was completely or partially unintelligible (Boucher, 2012; Kasari et al., 2014) and that speech reception was especially laborious if it contained many consonants, resulting in a speech that sounded like a sequence of vowels (McKeever et al., 2019). Furthermore, even when they have an excellent language level, individuals with ASD often exhibit atypical elocution (Nadig and Shaw, 2012), which presumably further denotes speech reception anomalies (Paul et al., 2005).

A large stream of recent studies shows that neural oscillations, i.e., the synchronous activity of neuronal populations, play a critical role in speech reception, mainly in parsing the speech flow into meaningful linguistic units: phonemes, syllables, words, phrases, etc. (Poeppel, 2003). While the 25- to 35-Hz low-γ oscillation rhythm is argued to work as a basic speech sampling rhythm enabling the encoding of phonemic-level acoustic features (Lehongre et al., 2011; Hyafil et al., 2015a; Hovsepyan et al., 2020), the 4- to 7-Hz θ rhythm (Giraud and Poeppel, 2012; Ghitza, 2013; Mai et al., 2016; Giraud, 2020) can flexibly track syllable boundaries, hence playing a key role in speech intelligibility (Hyafil et al., 2015a; Pefkou et al., 2017). On the other hand, the slower 1- to 3-Hz δ rhythm has an endogenous role in prosodic processing (Ghitza, 2017; Teoh et al., 2019) and phrasal chunking (Ding et al., 2014), while the 12- to 20-Hz low-β rhythm conveys top-down information, presumably at an intermediate timescale between phonemes and syllables (Pefkou et al., 2017; Giraud, 2020). Working in combination via their hierarchical coupling (the lower-frequency rhythm modulates the amplitude of higher-frequency activity), these different families of neural oscillations can underpin specific cognitive operations (Canolty and Knight, 2010; Hyafil et al., 2015b). In particular, θ/γ phase-amplitude coupling (PAC; Gross et al., 2013; Hyafil et al., 2015a,b; Lizarazu et al., 2019) enables the hierarchical encoding of the phonemic structure within syllables, while β/γ PAC is associated with specific operations in predicting and planning speech (Chao et al., 2018).

In typically developing (TD) children, the ability to track the speech temporal structure develops very early (Leong et al., 2017; Jessen et al., 2019; Attaheri et al., 2022; Cabrera and Gervain, 2020; Ortiz Barajas et al., 2021). The infant auditory system can already track the syllabic rhythm in nursery rhymes (Leong et al., 2017; Attaheri et al., 2022) and native language sentences (Ortiz Barajas et al., 2021). Even 1- to 8-d-old newborns can detect a consonant change on the sole basis of speech envelope cues (Cabrera and Gervain, 2020). Importantly, oscillation cross-frequency coupling (δ-θ/low-γ), which orchestrates the encoding of phonemic structure within syllables (Hyafil et al., 2015a), is found in infants younger than 11 months (Attaheri et al., 2022). All together, these findings suggest that the cortical tracking of speech rhythms is present at birth and likely contributes to phonological learning (Goswami, 2019). Its early disruption could be a possible cause of speech decoding impairment in children with ASD (Giraud and Poeppel, 2012; Jochaut et al., 2015; Menn et al., 2022), and would participate in the altered development of their social skills.

Oscillation anomalies in response to speech have previously been reported in ASD, such as decreased γ responses to rapid spectrotemporal transitions associated with diphones (Rojas et al., 2008; Gandal et al., 2010), increased θ responses to repeated tones and syllables (Wang et al., 2017; Zhang et al., 2018), and reduced β responses to novel sounds (Mamashli et al., 2017). Young adults with ASD (Jochaut et al., 2015) show joint anomalies of neural θ (syllabic-level) and low-γ (phonemic-level) activity, originating predominantly from the auditory cortex. In this brain region, the interplay between θ and γ neural activity was shown to be abnormal, suggesting that phonemic encoding was not temporally aligned with syllable tracking (Jochaut et al., 2015), a deficit that could deeply disrupt on-line speech reception (Hovsepyan et al., 2020). Although the observed θ/γ anomaly tightly correlated with the verbal scores of ASD participants (Jochaut et al., 2015), conclusions cannot be drawn on whether these oscillation coupling anomalies are specifically associated with the speech reception deficit. Furthermore, it is unknown whether these anomalies are present in very young children, when autistic symptoms usually become more evident, particularly during the development stage where typically developing children rapidly expand their speech repertoire (Press, 2015).

In the present study, we used high-density electroencephalography (EEG) to compare oscillatory neural processing of age-appropriate naturalistic speech in 64 very young children (1.31–5.56 years old) with and without ASD. Given the major developmental changes occurring in large-scale brain networks around this age in ASD (Nomi and Uddin, 2015), exploring speech reception as soon as the diagnosis is established is critical. If auditory cortical oscillations and their cross-frequency interactions are involved in language acquisition, we expect children diagnosed with ASD to already exhibit oscillation anomalies, notably in the δ-θ, low-β, and low-γ bands (Fujioka et al., 2012; Meyer et al., 2017; Pefkou et al., 2017; Molinaro and Lizarazu, 2018). We further expect these anomalies to specifically predict the language reception status (and not other developmental traits). The precise characterization of oscillation anomalies which are expected to be causally involved in speech reception difficulties is an indispensable first step to envisage possible targeted interventions aiming at normalizing (boosting or downregulating) oscillatory activity in ASD (Kayarian et al., 2020; Marchesotti et al., 2020).

## Materials and Methods

### Inclusion procedure

Sixty-four children (mean age: 3.00 ± 1.12, 17 females) with and without ASD drawn from the Geneva Autism Cohort (Franchini et al., 2018; Latrèche et al., 2021; Solazzo et al., 2021) underwent a large battery of tests, including EEG recordings while they watched movies (French cartoon aimed at young children: Trotro; Lezoray, 2013a,b,c,d). All children were recruited via specialized clinical centers or announcements in the community. The study was approved by the Ethics Committee of the Faculty of Medicine of the University of Geneva Hospital in accordance with the Declaration of Helsinki. A phone interview and a medical developmental history questionnaire were completed before the initial visit of each participant; for the typically developing children, the following were considered exclusion criteria: any suspicion of altered development, a history of neurologic or psychological disorder, and a family history of ASD in first-degree relatives. Parents gave their informed consent before inclusion in the study. The dataset selected for this study met the following criteria: the data were recorded during the participant’s first visit, the participant’s age was below six years old, the markers associated with movie onset were clear and accurate, there were exploitable raw data for four movies, the participant watched the screen during all the recordings. Given these strict inclusion criteria, we selected 64 children with and without ASD from the participants in the cohort.

For all ASD children, the clinical diagnosis was formally corroborated using the Autism Diagnostic Observation Schedule-Generic (ADOS-G; Lord et al., 2000) or the Autism Diagnostic Observation Schedule, second edition (ADOS-2; Lord et al., 2012). Data from 31 children with ASD (mean age: 3.09 ± 0.91) and 33 age-matched typically developing peers (mean age: 2.92 ± 1.30) were selected for further analyses (age difference: Kolmogorov–Smirnov D = 0.27, *p* = 0.19; see Tables 1, 2 and Extended Data Table 2-1 for participant characteristics). Table 1 summarizes the clinical characteristics of the ASD and the TD samples. Children with ASD scored significantly lower than their TD peers across all behavioral assessments, including language reception, language expression, visual reception, and fine motor skills.

**Table 1.** Participants’ demographic information and group comparison of behavioral tests

		Group							
		ASD (*N* = 31, 6 females)		TD (*N* = 33, 11 females)		Frequentist statistics		Bayesian independent samples *t* test	
		Mean	SD	Mean	SD	D	*p*	BF10	Error%
Age (in years)		3.09	0.91	2.92	1.3	0.62 (62)	0.54	0.3	0.011
Mullen Scales of Early Learning	Visual reception	87.18	26.37	126.13	27.28	−5.53 (56)	0.000000879	14940.11	2.21E-10
	Fine motor	82.42	21.29	103.22	12.01	−4.54 (56)	3.06E-5	624.4	1.48E-08
	Expressive language	58.52	31.69	104.77	24.08	−6.16 (55)	9.18E-08	117226.71	2.73E-11
	Receptive language	62.12	33.37	117.62	18.98	−7.71 (56)	2.33E-10	3.06E + 07	4.95E-13
Vineland adaptive behavior composite	Expressive language	10.94	3.55	17.15	2.14	−8.55 (62)	4.46E-12	1.23E + 09	5.29E-15
	Receptive language	11	2.8	16.27	2.11	−8.53 (62)	4.76E-12	1.31E + 09	4.69E-15

Mullen Scales of Early Learning (assessment of cognitive and motor strengths and weakness in children).

Visual reception and fine motor measure the child’s nonverbal ability to estimate the overall developmental level.

Expressive language and receptive language measure the child’s ability to process linguistic input and to use the language productively.

Vineland adaptive behavior composite (parent interview assessment of social, communication, motor, and daily living skills).

The scores are developmental quotient (DQ) score, that is ratio (%) of age equivalent by chronological age.

**Table 2.** Psychometric data of every participant

Group	Gender	Age	ADOS ALL	ADOS SSA	ADOS RRB	Symptom level	Mullen Scales of Early Learning												Vineland Adaptive Behavior Scales	
							Visual reception			Fine motor			Expressive language			Receptive language			Expressive language	Receptive language
							DQ	Raw	Tscore	DQ	Raw	Tscore	DQ	Raw	Tscore	DQ	Raw	Tscore		
ASD	M	2.54	7	6	7	Moderate	96.15	31	47	96.15	29	46	73.72	22	35	96.15	28	46	15	14
ASD	F	3.29	10	9	10	High	17.73	10	20	32.92	15	20	5.07	3	20	10.13	6	20	5	3
ASD	M	1.74	5	6	5	Moderate	82.63	19	36	82.63	18	34	46.48	10	21	67.13	14	26	10	13
ASD	M	2.76	10	9	10	High	90.75	25	34	77.3	22	20	50.41	15	24	53.78	15	20	10	8
ASD	F	2.25	7	8	5	Moderate	92.6	28	46	74.8	22	29	60.55	17	30	60.55	18	26	12	14
ASD	M	2.93	10	7	10	High	68.1	26	24	62.42	23	20	11.35	7	20	17.02	6	20	9	7
ASD	M	1.99	10	8	10	High	99.86	26	49	95.7	24	48	58.25	14	28	20.8	7	20	9	7
ASD	M	3.5	10	8	10	High	65.1	27	22	67.71	26	20	41.67	16	20	28.65	13	20	9	9
ASD	M	4.18	8	7	10	High	118.97	48	63	113.02	45	63	71.38	32	32	77.33	34	36	10	15
ASD	M	2.37	10	9	10	High	66.35	22	29	59.37	19	20	31.43	10	20	13.97	6	20	9	8
ASD	F	4.73	9	7	10	High	100.22	47	52	86.15	41	39	101.97	45	52	114.28	47	62	13	17
ASD	F	3.07	10	7	10	High	40.45	18	20	45.84	19	20	16.18	7	20	18.88	9	20	6	5
ASD	M	2.69	6	6	6	Moderate	80.53	28	39	71.24	24	26	40.27	13	20	43.36	15	20	9	10
ASD	M	3.01	6	4	10	Moderate	71.44	28	31	76.93	28	35	71.44	24	33	65.94	24	30	11	13
ASD	M	2.26	9	10	6	High	93.3	18	43	105.74	19	53	24.88	5	20	68.42	13	31	12	15
ASD	F	2.73	8	6	10	High	76.04	27	36	85.17	28	42	36.5	12	20	27.38	11	20	10	13
ASD	M	3.29	6	6	9	Moderate	83.65	33	40	76.04	29	32	55.76	21	22	63.37	25	27	10	10
ASD	M	3.35	8	7	10	High	111.73	41	55	84.42	32	36	89.39	32	43	81.94	30	38	13	8
ASD	M	2.01	7	6	9	Moderate	97.46	26	49	113.71	28	64	60.91	15	30	97.46	24	50	11	10
ASD	M	4.77	9	10	6	High	103.75	48	55	98.56	45	51	95.11	44	49	81.27	39	39	13	13
ASD	M	4.48	7	7	8	Moderate	83.72	41	37	115.34	47	65	78.13	36	37	85.58	38	41	13	14
ASD	M	2.72	6	4	9	Moderate	88.74	30	44	85.68	28	42	82.62	25	41	76.5	25	38	12	13
ASD	F	2.05	9	10	7	High	55.23	16	20	72.22	19	24	29.74	8	20	21.24	7	20	9	6
ASD	M	3.64	6	5	10	Moderate	130.55	47	69	96.2	37	46	125.97	44	64	107.65	39	54	19	15
ASD	M	2.66	4	3	8	Low	105.25	31	56	91.21	26	45	77.18	21	39	80.69	23	39	14	16
ASD	M	2.45	9	9	6	High	/	/	/	/	/	/	/	/	/	/	/	/	11	9
ASD	M	4.11	10	10	9	High	54.24	29	20	52.23	26	20	24.11	12	20	32.14	17	20	8	8
ASD	M	3.86	6	5	9	Moderate	147.49	50	80	121.84	45	69	123.98	45	63	126.11	45	65	15	14
ASD	M	1.99	7	9	5	Moderate	103.78	28	52	63.87	18	20	31.93	9	20	43.91	13	20	11	10
ASD	M	3.3	7	7	8	Moderate	77.8	32	37	67.76	27	23	45.17	18	20	70.27	27	34	9	9
ASD	M	5.14	4	4	7	Low	111.81	50	61	100.47	47	54	93.99	45	48	111.81	48	63	14	13
TD	M	1.98	1	1	1	/	102.62	27	53	90.31	23	44	73.89	18	38	82.1	21	40	16	15
TD	M	2.9	1	1	5	/	117.98			106.47			123.74			106.47			16	15
TD	M	4.15	1	1	5	/	131.99	49	68	97.99	41	48	119.99	46	62	123.99	46	65	17	22
TD	M	2.29	1	1	1	/	214.71	48	80	121.67	32	68	153.87	37	75	150.29	36	74	18	16
TD	M	2.22	1	1	1	/	123.01	33	61	104.37	28	53	134.19	32	66	123.01	30	59	19	19
TD	F	2.65	1	1	5	/	141.25	41	72	103.59	31	53	131.84	36	67	128.7	35	65	18	16
TD	F	1.34	1	1	1	/	180.75	30	80	112.19	20	49	124.66	19	54	155.82	25	69	17	15
TD	M	1.73	1	1	1	/	144.15	31	79	86.49	20	43	129.74	25	68	144.15	28	72	19	17
TD	M	3.12	1	1	1	/	144.84	46	75	91.2	32	42	123.38	39	62	109.97	35	54	17	21
TD	M	1.8	1	1	1	/	115.92	27	58	120.55	26	62	69.55	15	33	88.1	20	42	13	15
TD	F	1.8	2	2	1	/	125.19	29	64	120.55	26	62	69.55	15	33	115.92	25	58	9	15
TD	M	5.44	1	1	1	/	101.18	49	48	104.25	49	53	102.72	48	51	105.78	48	54	15	16
TD	M	2.34	1	1	6	/	103.29	30	53	92.6	26	45	124.66	31	64	117.54	30	59	17	17
TD	M	2	1	1	1	/	/	/	/	/	/	/	/	/	/	/	/	/	14	16
TD	F	2.19	1	1	1	/	/	/	/	/	/	/	/	/	/	/	/	/	16	16
TD	M	2.67	1	1	1	/	109.5	35	58	100.38	31	53	109.5	32	58	139.92	38	70	17	18
TD	M	4.76	1	1	1	/	/	/	/	/	/	/	/	/	/	/	/	/	17	21
TD	M	1.7	1	1	1	/	/	/	/	/	/	/	/	/	/	/	/	/	16	14
TD	M	5.33	1	1	1	/	107.02	50	69	79.11	42	46	108.58	50	75	96.17	46	61	17	18
TD	M	3.14	1	1	5	/	112.27	40	58	109.66	37	59	117.49	38	60	122.71	39	63	17	16
TD	M	5.1	1	1	1	/	114.94	50	58	113.27	49	58	96.61	45	45	98.28	45	46	16	20
TD	F	2.73	1	1	5	/	/	/	/	/	/	/	/	/	/	/	/	/	17	14
TD	M	5.56	1	1	1	/	98.84	49	48	101.84	49	53	79.37	43	39	92.85	46	44	17	19
TD	M	3.82	1	2	1	/	143.91	49	79	115.56	43	68	/	/	/	124.28	44	67	17	19
TD	F	1.57	1	1	1	/	92.02	21	48	102.24	21	54	71.57	14	37	122.69	24	67	12	15
TD	F	1.31	1	1	1	/	104.89	20	53	92.55	17	42	129.56	20	66	117.22	20	60	17	17
TD	F	2.73	1	1	1	/	123.6	39	66	93.45	30	49	87.42	27	46	117.57	34	62	20	17
TD	M	4.68	1	1	1	/	116.92	49	60	93.89	43	45	93.89	43	46	97.43	43	48	18	18
TD	F	1.87	1	1	1	/	138.26	32	75	120.42	27	66	75.82	17	39	124.88	27	65	15	19
TD	M	2.23	1	1	5	/	107.61	31	56	96.85	27	48	82.5	22	41	107.61	28	53	15	15
TD	M	1.68	1	1	1	/	172.07	34	80	121.46	25	64	86.04	17	43	151.83	28	72	15	17
TD	F	3.13	1	1	5	/	127.73	43	65	87.82	31	39	87.82	33	49	109.11	35	54	15	20
TD	F	4.33	1	1	1	/	115.07	48	59	109.32	45	56	120.82	47	61	118.91	46	61	18	18

See Extended Data Table 2-1 for psychometric data of every participant.

10.1523/JNEUROSCI.0112-22.2023.t2-1 Extended Data Table 2-1 Psychometric data of every participant. The Table was organized for easily download. Download Table 2-1, XLSX file.

### Cognitive skills measure

Developmental functioning was assessed using the Mullen Scales of Early Learning (MSEL; Mullen, 1995), which comprise five subscales, namely gross motor, visual reception, fine motor, receptive language, and expressive language. The four latter scales are so-called “cognitive scales” and are used to derive an Early Learning Composite score as a measure of overall developmental functioning. The subscales of visual reception and fine motor skills measure nonverbal ability, while receptive language and expressive language measure the ability to process linguistic input and use the language productively. The subtest raw scores were converted into age-adjusted normalized developmental quotient (DQ) scores, obtained by dividing the age-equivalent scores by the child’s chronological age and multiplying the result by 100.

The communication skills assessment, i.e., receptive language and expressive language, was completed by the Vineland Adaptive Behavior Scales, second edition (VABS-II; Sparrow et al., 2005), testing of adaptive function that does correlate with cognition. VABS-II is a parent interview which assesses social, communication, motor, and daily living skills, and provides age-equivalent and standard scores for a variety of summary scales and subscales, including expressive and receptive language and social adaptive functioning. Age-equivalent scores are reported.

### Symptom severity

Three symptom severity levels, i.e., low, moderate, and high, were based on ADOS-G (Lord et al., 2000) or the ADOS-2 (Lord et al., 2012) calibrated severity scores (Table 2), which varied from 3 to 10 distributed into three severity grades: 3–4, low level; 5–7, moderate level; 8–10, high level.

### Stimuli and procedure

**Figure 1.**
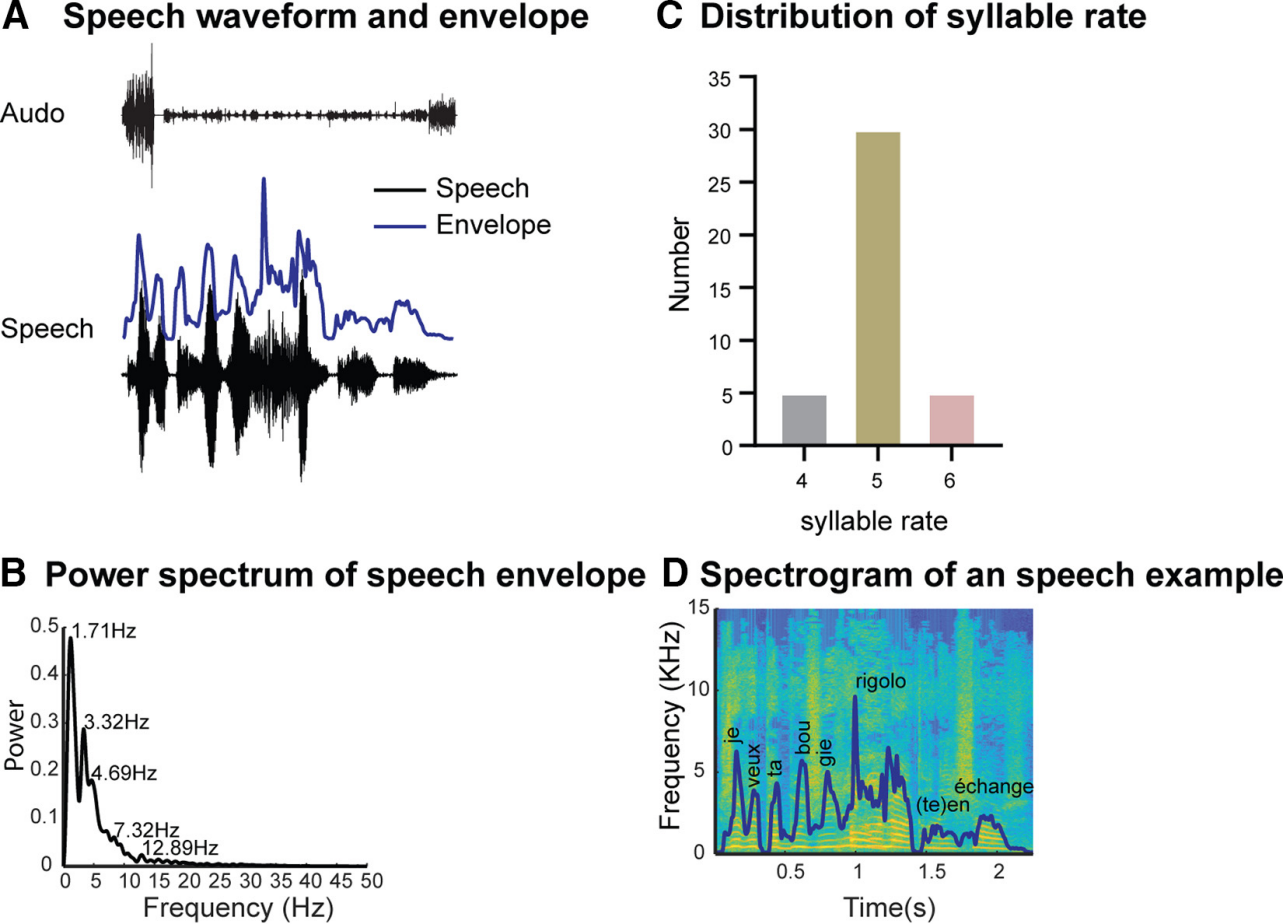
Stimulus properties. ***A***, Speech waveform and envelope. In black: original audio track and waveform; in blue: speech envelope. ***B***, Power spectrum of speech envelope. The average power spectrum across all speech samples shows dominant frequencies between 1 and 7 Hz, with 1.17-, 3.32-, and 4.69-Hz peaks. ***C***, Distribution of syllable rate across all speech samples. ***D***, Spectrogram of a speech sample, the sentence “Je veux ta bougie rigolote en échange” (I want your funny candle in exchange). Also, see Extended Data Figure 1-1 for preprocessing pipeline.

We explored cortical speech processing during a passive, naturalistic task with a relatively low cognitive demand suitable for young children. Participants watched four French Trotro (Lezoray, 2013a,b,c,d) cartoon videos, each lasting ∼2.5 min. The video presentation was controlled by Tobii Studio (Tobii Technology, Sweden). The screen size was 1200 pixels (29°38′) in height and 1920 pixels (45°53′) in width, with a 60-Hz refresh rate. Participants were seated ∼60 cm away from the screen. The cartoon soundtrack was delivered via loudspeakers at a sound level adjusted for each participant. As the sound level was not monitored during the experiment but only set to a comfortable level, we cannot exclude sound level differences across groups. The soundtrack sampling rate was 44.1 kHz. We removed background noise, e.g., birds’ singing, and music, from the original movie soundtrack using Audacity v.2.2.1 (Audacity Team, 2021) editing software (Fig. 1; Extended Data Fig. 1-1). We then extracted the stimuli envelopes using the absolute value of the analytic signal (Boashash, 2015). The speech envelope was down-sampled to 1000 Hz, and low-pass filtered using a zero-phase fourth-order Butterworth filter set at 40 Hz (Fig. 1*A*). An envelope spectral decomposition was performed using the fast Fourier transform (Fig. 1*B*). We found dominant frequencies between 1 and 7 Hz, with peaks at 1.17, 3.32, and 4.69 Hz, overlapping with the syllable rate range (four to six syllables per second; Fig. 1*C*) as determined by averaging peaks within the 150-ms minimum-peak-distance that were associated with the averaged French-syllable duration (Pellegrino et al., 2011). The spectrogram of the example sentence: “je veux ta bougie rigolote en échange” (I want your funny candle in exchange), shown in Figure 1*D*, was calculated using the MATLAB function “spectrogram” (The MathWorks).

10.1523/JNEUROSCI.0112-22.2023.f1-1 Extended Data Figure 1-1 Stimulus properties and schematic of the processing pipeline. There were four main steps of the processing: stimulus presentation and EEG and eye-tracking data recording (step 1), preprocessing of stimuli (step 2.1, extracting speech envelope, steps 2.2 and 2.3 eye-tracking and EEG data preprocessing), data processing based on purpose (step 3 left up, neural tracking via multiple linear regression model; step 3 right up, frequency decomposition via wavelet transformation and phase-amplitude coupling via KL-MI-Tort approach; step 3 bottom, prediction of clinical variables via LASSO); and last step, step 4, between-group statistical analysis. For detailed information on each step, please see Materials and Methods. Download Figure 1-1, TIF file.

### EEG acquisition and preprocessing

EEG data were acquired using a Hydrocel Geodesic Sensor Net (HCGSN, Electrical Geodesics) EEG system with 129 scalp electrodes at a 1000-Hz sampling rate. The recording reference electrode was located on the vertex (Cz), and a real-time bandpass filter at 0–100 Hz was applied to the incoming signal. The first two cartoon videos were presented in the first block, and the last two cartoon videos in a second block, with a ∼5 min “dynamic image” task (Sperdin et al., 2018) in between cartoon videos and a 10-min break between blocks. At the end of the first block, the electrode impedances were measures, and if needed, the electrodes’ conductance was adjusted by applying conductive gel to keep impedances below 40 kΩ.

The EEG signal preprocessing was conducted using the EEGLAB v2019 toolbox within the MATLAB environment (Delorme and Makeig, 2004) and Cartool (https://sites.google.com/site/cartoolcommunity/). First, the dataset underwent a dimensionality reduction to 110 channels by excluding cheek and neck electrodes that are often contaminated by muscle artifacts. Then, a zero-phase fourth-order Butterworth bandpass filter between 0.1 and 70 Hz was applied to the EEG signals, as well as a notch filter at 50 Hz to remove power line interference. Each participant’s dataset was then visually inspected to exclude periods contaminated by movement artifacts. An independent component analysis (ICA) was computed on the dataset to identify and remove components with eye blinks, saccades, electrical line noise, and heartbeat artifacts. The dimensionality of the components was equivalent to the number of electrodes kept after bad channel detection. ICA was performed in MATLAB and Cartool. The first step was the detection of bad channels, i.e., with too large signal amplitude because of defective electrodes. The remaining data were decomposed into independent components using the Runica algorithm (EEGLAB function). Based on the ICA components’ time course and topographies, noisy components were visually determined and excluded.

Subsequently, a spherical spline interpolation was used to interpolate the channels contaminated by noise using the ICA-corrected data. Finally, the Cartool spatial filter (Michel and Brunet, 2019) was applied, and a common average reference was recalculated on the cleaned data. The spatial filter is an instantaneous filter that removes local outliers by spatially smoothing the map without losing its topographical characteristics. It was run in the following way: (1) for each electrode, the values of the 6 closest neighbors are determined, plus the central electrode value itself; (2) the seven data points are sorted; (3) the minimal and maximal values are removed by dropping the first and last items; (4) the remaining five values are averaged, with weights proportional to the inverse distance to the central electrode, which is given a weight of 1 (Michel and Brunet, 2019).

A trial was defined based on the beginning and end of each speech chunk in the cartoon, leading to a total of 50 trials with an average duration of 1.69 s. The detected artifact periods were replaced by NaNs (not a number). After excluding artifact time segments, fewer trials remained in the ASD group than in the TD group (unpaired *t* test comparison *t*_(62)_ = −2.54, *p* = 0.014). There were 35.12 ± 1.88 (mean ± SD, range 28–37) trials in the TD group, against 32.97 ± 4.48 (mean ± SD, range 21–36) in the ASD group. The mean trial duration was similar across groups (unpaired *t*_(62)_ = 0.98, *p* = 0.33). The mean trial duration was 2454.4 ± 92.15 ms (mean ± SD) in the ASD group and 2431.8 ± 93.02 ms (mean ± SD) in the TD group.

### Eye-tracking acquisition and analysis

To assess whether the observed effects were because of differences in the way participants explored the visual scene (gaze focus differences), we collected gaze data using a Tobii TX300 eye-tracking system (https://www.tobiipro.com) with a 300-Hz sampling rate. The cartoon frames allowed for a visual angle of 26°47′ × 45°53′ (height × width). A five-point calibration procedure consisting of child-friendly animations was performed using an inbuilt program in the Tobii system. The calibration procedure was repeated if the eye-tracking device failed to detect the participant’s gaze position accurately. The lighting conditions in the testing room were constant for all acquisitions. Younger participants sat on their parent’s lap to make them feel comfortable and minimize head and body movements. All participants watched all four cartoon videos in the same order. To extract fixations, we used the Tobii IV-T Fixation filter (Olsen, 2012; Kojovic et al., 2022).

### Gaze divergence estimation

**Figure 2.**
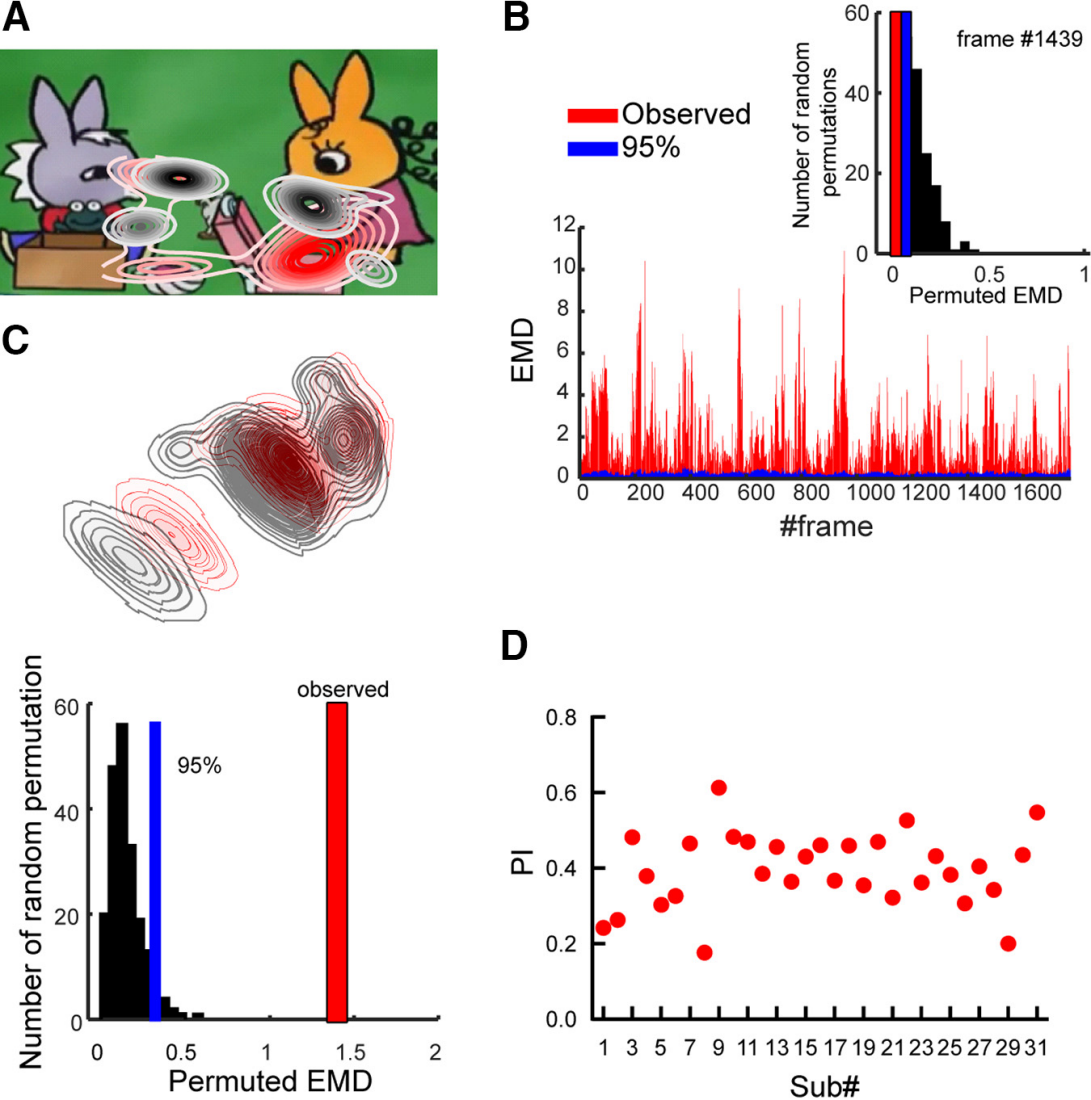
Gaze divergence estimation. ***A***, An example frame with gaze distribution in TD (delimited by black contours) and ASD (red contour) children. ***B***, Top, Example of distribution of EMD in one frame. (bottom) EMD values in all frames. Red: observed EMD; blue: the 95% confidence interval (CI) of the null distribution. If the observed EMD is bigger than 95% CI, the gaze distribution is significantly different across groups. ***C***, Top, Cumulative gaze distribution (red contour: ASD, black contour: TD) over all frames and all speech excerpts. Bottom, Distribution of the permuted EMD (black histogram) and the observed EMD (red bar) of the contours in ***C***, top. ***D***, Mean Proximity Index (PI) values for all 31 children with ASD and all speech excerpts.

To assess potential group differences in visual exploration, we first followed Kojovic et al. (2022) approach to create a gaze distribution map for each frame of the movie(s). A Gaussian kernel (adaptive bandwidth) was applied to each pair of gaze coordinates, and the results were added to obtain an estimation of gaze density (Botev et al., 2010). This step was run with the MATLAB function *akde*. The probability of gaze allocation at a given point of the visual scene was represented by the obtained density estimation (Fig. 2*A*). Next, we used the earth mover’s distance (EMD; Rubner et al., 2000; Rohrbein et al., 2015; Cho et al., 2016; Orlova et al., 2016; Yilmaz, 2021), known in mathematics as the Wasserstein metric, to measure between-group differences in gaze distribution in terms of location, i.e., *x-y* grid, and frequency, i.e., the obtained density. To address whether gaze patterns statistically differed between the ASD and TD groups, we used a permutation test. First, we shuffled the labels for the ASD and TD groups to create a null distribution for the earth mover’s distance (EMD), a measure of the distance between the two gaze distributions. We repeated this process 200 times to obtain an empirical null distribution for the EMD. It is important to note that two distinct types of distributions are being referred to here: the distributions of gaze for the ASD and TD groups, and the empirical null distribution of EMDs between these two distributions. To compute the one-tail probability, we ranked the observed EMD within the empirical null distribution. A rank >195 (top 5%) within the 200-element null distribution was considered significant. The approach was applied frame-by-frame to obtain single frame gaze-distribution difference (Fig. 2*B*), and a cumulative gaze-distribution over frames in the whole speech excerpt (Fig. 2*C*, top) and the cumulative gaze-distribution difference (Fig. 2*C*, bottom). Although gaze distribution did not significantly differ between groups within each frame (Fig. 2*B*), a significant effect was found on the cumulative gaze distribution (Fig. 2*C*); the observed EMD (red bar, i.e., observed EMD = 1.39 in Fig. 2*C*) is distinct from the 95% of the null distribution (Fig. 2*C*, blue bar). We then assessed individual gaze divergence in ASD by calculating the distance between every ASD gaze and the TD gaze norm on each frame. For comparison and interpretation, the distance was normalized to 0–1 and named Proximity Index (PI; Kojovic et al., 2022). A high PI value denotes that the visual exploration of an individual for a given frame is closer to the norm (more TD-like). A summary measure of divergence in visual exploration from the TD group was obtained by averaging PI values over all frames in each speech excerpt (Fig. 2*D*). Figure 2*D* indicates that for every child with ASD, the gaze pattern diverges (PI) from the TD group to a different extent. The PI values were included as covariates to reduce the gaze pattern bias in EEG signals.

### EEG time-frequency analysis

To identify the oscillatory responses to speech, a Morlet wavelet transformation was performed from 5 s before to 5 s after each speech chunk (making sure the epoch would not include any artifacts) onset between 0.1 and 50 Hz with five cycles for Gaussian taper at each EEG electrode. Then time course between 1 s preonset to 1 s postonset was selected (based on those speech chunks with the shortest duration) on a trial-by-trial basis, and the power was averaged across trials and normalized by decibel conversion (dB) over a −1000- to 0-ms baseline period (for which an audio stimulus was presented but no speech), allowing for between-group comparisons. EEG power in the different frequency bands of interest was defined as the mean power value across 0–1000 ms postspeech stimulus onset. We compared ASD and TD groups in four frequency bands that are relevant for speech processing, i.e., δ (1–3 Hz), θ (4–8 Hz), β (12–20 Hz), and low-γ (25–35 Hz).

### Speech envelope prediction from EEG power modulation: multiple linear regression model (MLR) with distributed lags

**Figure 3.**
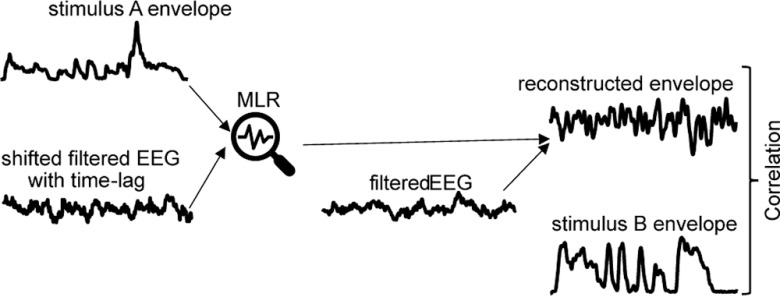
Schematics of the multiple linear regression (MLR) model approach. The filtered EEG signal and stimulus envelope were entered into the MLR. For the cross-validation procedure, envelopes of speech and EEG signal of 9-fold with time-lag shifting were used to fit an MLR for each participant, which was then used to predict the envelope of the 10th speech fold. The resulting model was tested by correlating the predicted envelope with the actual speech envelope in the test segment.

To probe differences between ASD and TD in oscillatory speech tracking, a multiple linear regression (MLR) model with a distributed lag between −300 and 300 ms with 50-ms steps (Fig. 3) was used to reconstruct the stimuli envelopes from the neural responses. The EEG signals were lagged to compensate for possible differences in temporal alignment between the brain response and the stimulus, a widely used method in the literature (Crosse et al., 2016; Di Liberto and Lalor, 2017; Fiedler et al., 2017; Di Liberto et al., 2018). A multiple linear regression model was trained on the resulting set using a 10-fold cross-validation approach. First, all speech chunks were divided into ten consecutive segments. In each fold, one segment was left out for testing and the remaining segments were used for training. This process was repeated 10 times, ensuring that each segment was only used once for testing. On each fold, a multiple linear model was used to find a linear combination among the brain signals that best predicted the time course of the speech envelope. The resulting model was tested by correlating the predicted envelope with the actual speech envelope in the test segment. The final result, representing the oscillation tracking index, was obtained by averaging Fisher’s *z*-transformed correlations in each fold and then taking the inverse Fisher's transformation of the resulting mean *z*-score. The model was trained separately for each individual subject.

We deployed the approach separately in the two frequency bands implicated in speech tracking, i.e., θ and δ, and computed the amplitude of specific bands to obtain the oscillation tracking index. The amplitude was the absolute value of the Hilbert transform in band-specific filtered EEG, i.e., 4–8 Hz for θ and 1–3 Hz for δ.

### Phase-amplitude coupling

The time-courses from electrode clusters selected from the EEG power and neural tracking analyses were examined for phase-amplitude coupling changes (PAC). The first step was applying the Hilbert transform to each bandpass Butterworth filtered trial (Le Van Quyen et al., 2001) to obtain low-frequency phase (*f*_p_) and high-frequency amplitude (*f*_a_). Considering the filters for extracting *f*_a_ must be wide enough to capture the center frequency ± the modulating *f*_p_ to detect PAC (Dvorak and Fenton, 2014; Aru et al., 2015), we decided to use a variable bandwidth, defined as center frequency ± the modulating center frequency, to improve PAC detection. The *f*_p_ bandwidth was kept narrow (center frequency ± 1Hz) to extract sinusoidal waveforms. Furthermore, changes in the modulation power spectrum between speech and baseline periods were visually inspected in each participant to confirm that oscillation peaks/troughs were present at each modulating frequency-band of interest *f*_p._ For instance, if the interested modulating frequency band was 4–8 Hz, we confirmed that participants presented a real peak in the power spectrum at 4–8 Hz. Next, the coupling between *f*_p_ and *f*_a_ was quantified using the Kullback–Leibler modulation index (Tort et al., 2010). The KL-MI-Tort approach estimates PAC by quantifying the amount of deviation in amplitude-phase distributions. That is breaking *f*_p_ into 18 bins, and then calculating the mean amplitude within each phase bin, finally normalized by the average value across all bins. The modulation index is calculated by averaging the distance of the observed amplitude-phase distribution (*P*) and a uniform amplitude-phase distribution (*Q*).

$$MI=\frac{D(P,Q)}{log(N_{bin})}.$$

Mathematically, the Kullback–Leibler distance (*D*) was computed using the following formula (Seymour et al., 2017).

$$D\left(P,Q\right)=\overset{N}{\underset{i_{bin}=1}{\sum }}P(i_{bin})\cdot \log\frac{\left(P(i_{bin}\right)}{Q(i_{bin})}).$$

Using KL-MI-Tort, we calculated PAC between phases 2–15 Hz (in 1-Hz steps) and amplitudes 16–50 Hz (in 2-Hz steps) for the time period 0–1000 ms following speech onset and a 1000 ms prestimulus baseline period. MI values were calculated separately for each trial and averaged to obtain a single MI value per amplitude and phase. To normalize MI values, this was repeated using surrogate data, created by shuffling trial and phase-carrying information (200 surrogates).

### Predicting clinical variables from oscillatory features

In order to show the relationship between the brain activity and the neurophysiological variables, we tested whether autism severity was predicted by using only band-specific power per electrode (i.e., δ, θ, β, and low-γ power), or only neural tracking values per electrode, or only phase-amplitude coupling matrices per cluster (i.e., maximum MI value and corresponding phase-frequency, amplitude-frequency). For this, we trained a Linear Discriminant Analysis (LDA) classifier using a 10-fold nested cross-validation procedure, which separates the data into test and training sets. The training set was further separated using a 5-fold cross-validation approach for parameter search (Grid search). The LDA classifier was trained using a diagonal shared covariance matrix. The cross-validation process ensured that the training and testing datasets were not overlapping, avoiding misleading results because of overfitting. The input to the classifier was the band-specific EEG power, neural-tracking, and PAC for each participant, and the label to be predicted was symptom severity (i.e., low, moderate, and high). All empirical thresholds were obtained through cumulative binomial distribution (Combrisson and Jerbi, 2015).

Then, we probed the relationship between the neural and cognitive skills using a regularized linear model per frequency band (i.e., δ, θ, β, and low-γ power), per neural tracking per electrode, and per PAC matrix (i.e., maximum MI value and corresponding phase-frequency, amplitude-frequency) to determine which critical oscillation (or combination of oscillations) was the best predictor of cognitive skills within group, e.g., the higher the tracking value, the higher the speech reception. The linear model was based on Lasso regression which requires finding the best hyper-parameters within high dimensional data (Tibshirani, 1996). We also used a 10-fold nested cross-validation approach to improve model selection. The training set was further separated using a 5-fold cross-validation approach for parameter search (Grid search). The results were presented as averaged *R*^2^ values, which indicate the prediction power of a given feature, i.e., higher *R*^2^s signal higher prediction accuracy.

### Statistical analysis

Between-group statistical comparisons of band-specific EEG power and neural tracking were done using cluster-based nonparametric permutation tests with Monte Carlo randomization (Maris and Oostenveld, 2007) using the FieldTrip toolbox (Oostenveld et al., 2011; http://fieldtriptoolbox.org) with gaze divergence as a covariate to remove any possible bias because of gaze differences between groups. In detail, between-group differences (ASD vs TD) were assessed with unpaired t-statistics. First, clusters of significant group differences were obtained by considering at least two adjacent electrodes whose *t* value exceeded a 5% significance threshold (ASD vs TD, unpaired *t* test, uncorrected for multiple comparisons). The maximum *t* value within each cluster was carried forward. Next, a null distribution was obtained by randomizing the gaze divergence label 1000 times and calculating the largest cluster-level *t* value for each permutation. The maximum *t* value within each original cluster was then compared against this null distribution, with values exceeding a threshold of *p *<* *0.05 deemed significant.

In addition, between-group differences of neural tracking were tested by means of unpaired *t* test for all electrodes collectively analyzed. For group comparison statistics, only one value of θ-/δ-neural tracking per participant was included in the unpaired *t* test comparing ASD and TD groups.

To assess changes in the comodulograms of PAC between the speech and baseline periods, we processed the nonparametric cluster-based statistics (Maris and Oostenveld, 2007). First an uncorrected dependent-samples *t* test was performed (speech vs baseline), and then MI values exceeding a 5% significance threshold of null distribution were grouped into clusters.

## Results

### Speech-related oscillatory changes

**Figure 4.**
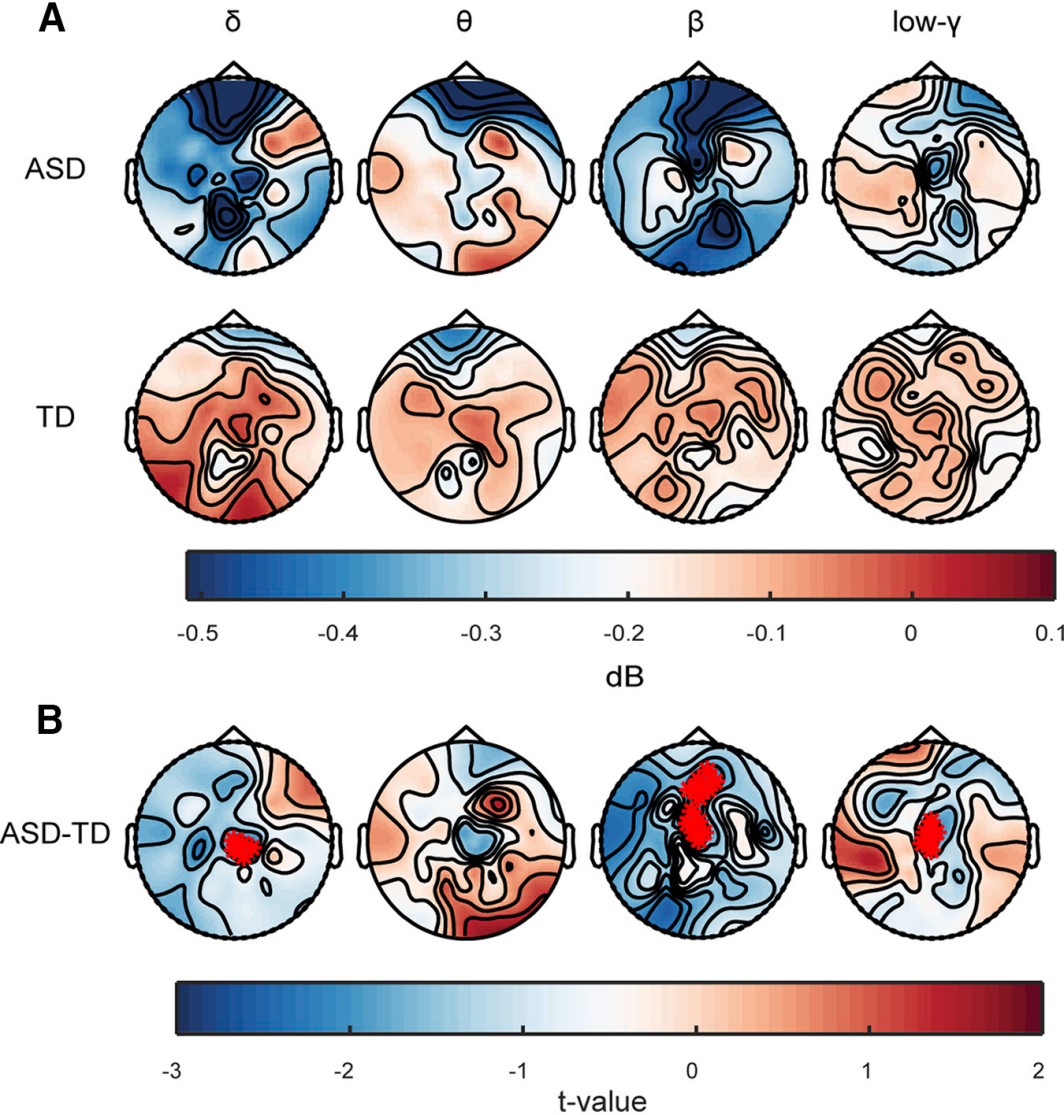
Speech-related oscillation changes. ***A***, Scalp topographies of power in several frequencies of interest in ASD and TD groups. ***B***, Comparison of frequency-power among 31 children with ASD and 33 TD peers. Children with ASD had reduced δ, β, and low-γ power and comparable θ power relative to their TD peers. Asterisks in scalp topographies indicate group differences after cluster correction (cluster-based nonparameters permutation tests, cluster corrected *p* = 0.05). Also, see Extended Data Figure 4-1 for a comparison of α frequency-power in both groups.

To analyze EEG power and possible probe differences in neural activity in response to speech across groups (Fig. 4*A*), we computed the EEG power spectrum of speech compared with baseline in several frequency bands of interest, i.e., δ (1–3 Hz), θ (4–8 Hz), low-γ (25–35 Hz), and β (12–20 Hz). A between-group statistical comparison (Fig. 4*B*; Extended Data Fig. 4-1), with gaze divergence as a covariate, showed reduced δ, β, and low-γ band oscillatory activity (mostly on mid-central clusters) in children with ASD. In contrast, θ oscillations were comparable to their TD peers.

10.1523/JNEUROSCI.0112-22.2023.f4-1 Extended Data Figure 4-1 Comparison of α frequency power in 31 children with ASD and 33 TD peers. Children with ASD had comparable α power relative to their TD peers (cluster-based nonparameters permutation tests, cluster corrected *p* = 0.05). Download Figure 4-1, TIF file.

### Neural tracking of speech envelope using multiple linear regression model

We then explored the differences between ASD and TD in the neural tracking of the cartoon soundtrack’s phrase-level and syllable-level modulations. The full segment duration differed across participants and across groups. The mean duration was 80,935 ± 11 524 ms (mean ± SD) in the ASD group, and 85,653 ± 6712.7 ms (mean ± SD) in the TD group. The model was trained by a 10-fold cross-validation approach, meaning that the duration of each fold was around 8093.5 ms for ASD and 8565.3 ms for TD. The duration in both groups was enough to get a valid value. Please note that the speech content is not expected to affect low-frequency neural tracking. Therefore, although the balance across groups was not perfect, the result remains reliable.

**Figure 5.**
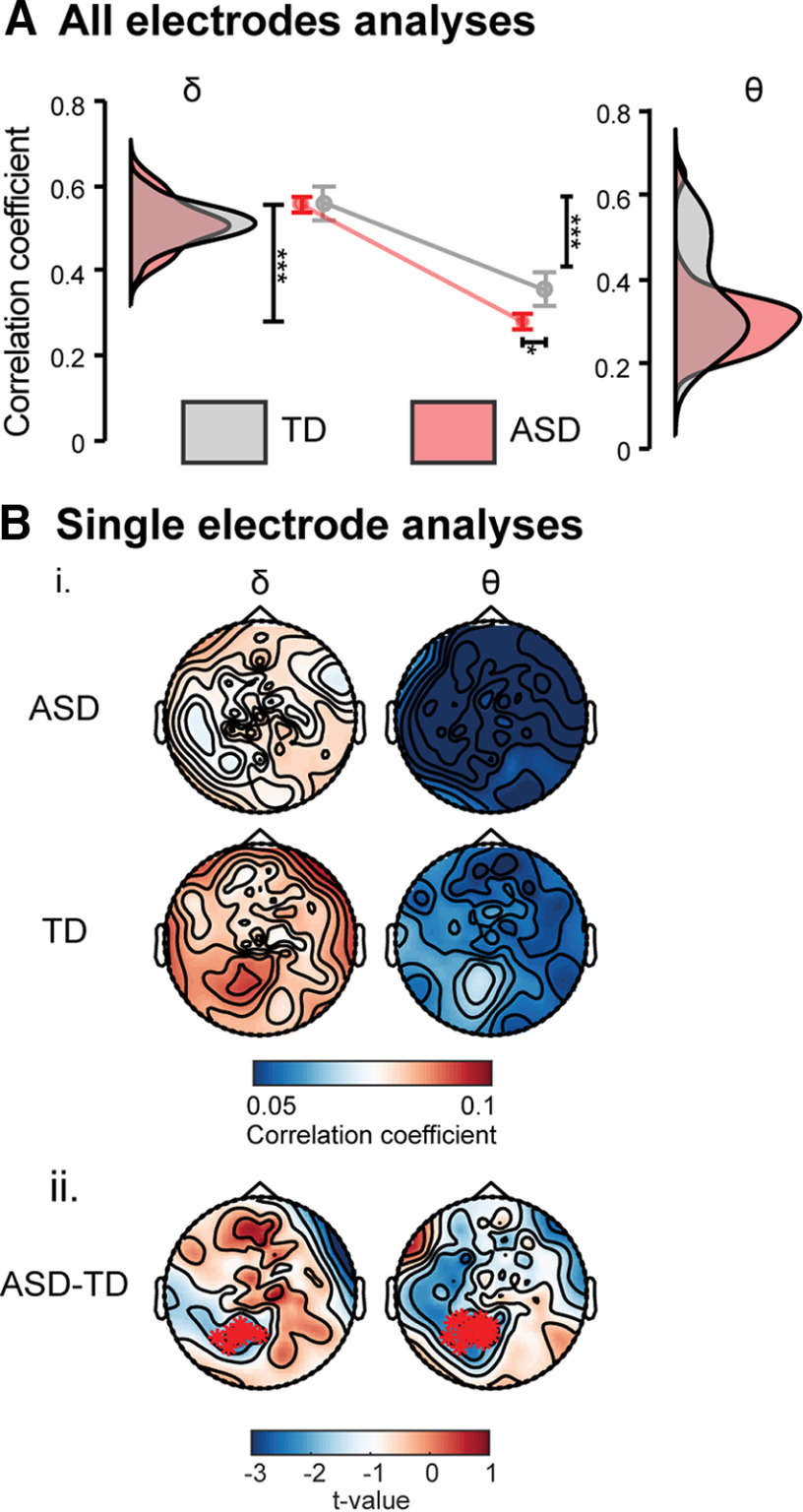
Neural tracking of speech envelope. Correlation coefficients between the reconstructed and real envelope, in all channels together (***A***) and each single-channel separately (***B***). ***Bi***, Correlation coefficient topographies in both groups. ***Bii***, Correlation coefficients were significantly reduced in ASD relative to TD group in the θ band. Error bars in ***A*** represent standard error (SE) of correlation coefficients and shadows in ***A*** represent the neural tracking distribution in each group. Asterisks in ***B*** show group differences from nonparametric cluster-based permutation tests, *p < 0.05, **p < 0.01, ***p < 0.001.

We found that the speech envelope reconstruction was significantly less accurate in ASD participants using the θ band signal (unpaired *t*_(62)_ = 2.19, *p *=* *0.03, η^2^ = 0.07; Fig. 5*A*). This θ effect was most prominent in a specific cluster of 12 posterior-occipital electrodes (Fig. 5*B*). The δ-band signal from all electrodes permitted the reconstruction of the stimulus envelopes equally well in ASD and TD participants (unpaired *t*_(62)_ = 0.18, *p *=* *0.86, η^2^ = 0.0005; Fig. 5*A*). However, we found reduced speech envelope reconstruction accuracy from the δ-band signal in ASD participants relative to TD participants in a specific cluster of seven parieto-occipital electrodes (Fig. 5*B*). These results denote that although the overall θ power was unchanged in children with ASD (Fig. 4), the neural tracking of the speech syllabic structure by δ-range and θ-range neural activity was altered.

### Phase amplitude coupling

**Figure 6.**
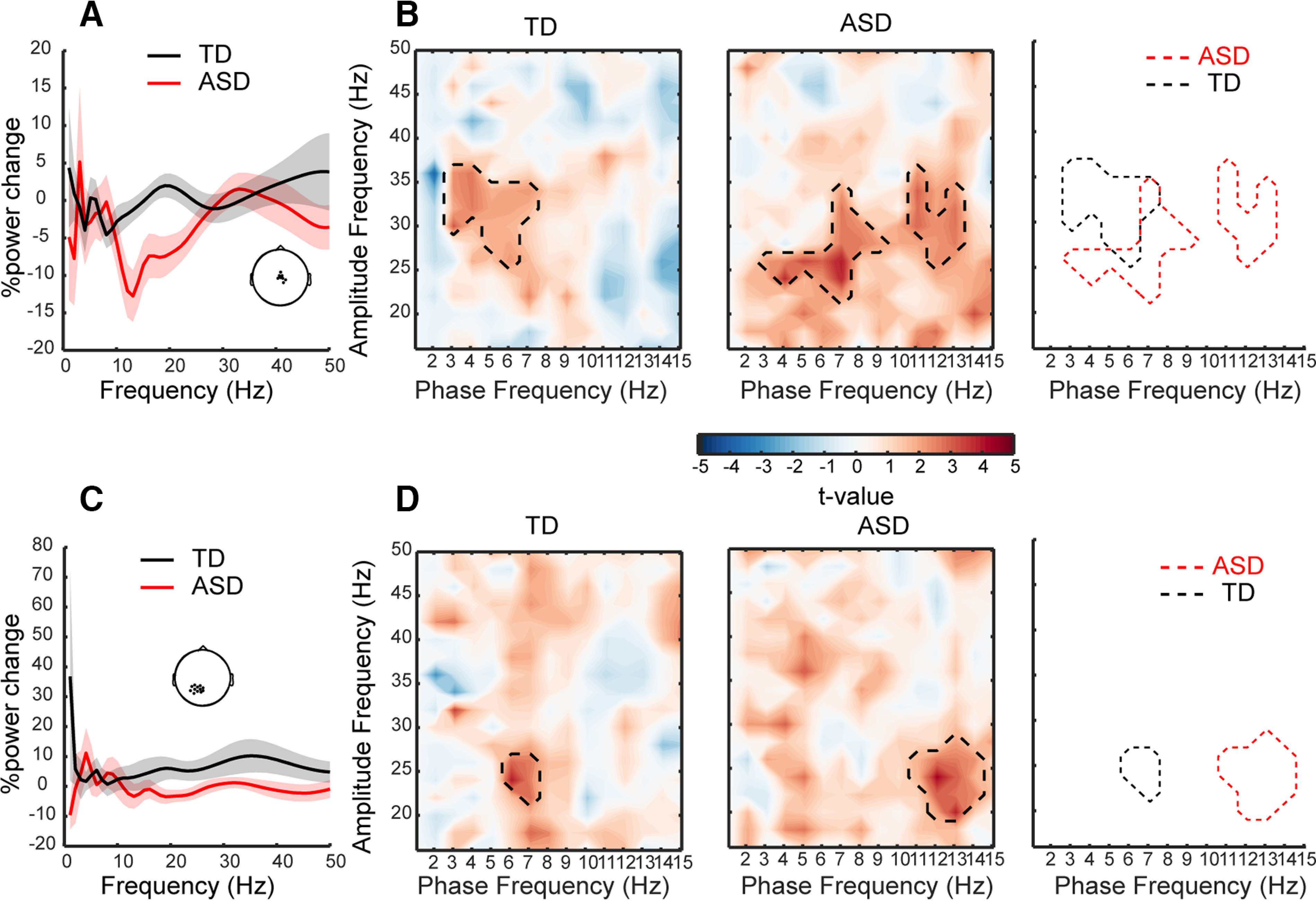
***A***, ***C***, Speech-induced oscillatory power is characterized by a strong power peak in low-frequency bands (1–10 Hz) in both groups and a marked power trough in the low-β band (10–15 Hz) in the ASD group over (***A***) central electrodes selected from EEG power group differences and (***C***) posterior-occipital electrodes selected from neural tracking group differences. ***B***, ***D***, Phase-amplitude comodulograms produced by statistically comparing the coupling values represented by modulation index (MI) values in the speech and baseline periods over central electrodes (***B***) and posterior-occipital electrodes (***D***). Dotted lines represent significant differences in phase-amplitude coupling. For exact cluster locations, see topographies on ***A***, ***C***. For a quick appraisal of *f*_p_ and *f*_a_ ranges in each group, see the rightmost panel (*f*_p_: frequency of phase; *f*_a_: frequency of amplitude, nonparametric cluster-based statistics, cluster-corrected *p* < 0.05). Also, see Extended Data Figures 6-1 and 6-2 for individual speech-induced oscillatory power in both groups.

As speech is encoded hierarchically by different families of nested neural oscillations, we also analyzed phase-amplitude coupling (PAC) across modulating (<15 Hz) and modulated (16–50 Hz) frequencies using the KL-MI-Tort. This approach was applied to those clusters showing significant between-group differences in the EEG power (all electrodes of clusters in the mid-central area; see Fig. 4) and in the neural tracking analyses (θ-tracking cluster; Figure 4*B*). Before computing PAC and assessing PAC changes, we checked that there were clear power spectrum peaks and troughs at each modulating frequency band of interest (Dvorak and Fenton, 2014; Aru et al., 2015). Despite large interindividual variability in peak/trough frequencies and oscillatory power, average neural activity confirmed significant power peaks/troughs in the low-frequency range in both groups (Fig. 6*A*,*C*; Extended Data Figs. 6-1, 6-2), enabling us to compute comodulograms for speech and baseline EEG, and to compare them using cluster-based nonparametric statistics. Note that the potential effects of gaze patterns were considered in the statistical analyses and removed. The reported effects hence primarily denote auditory processes.

10.1523/JNEUROSCI.0112-22.2023.f6-1 Extended Data Figure 6-1 Individual speech-induced oscillatory power in the TD group. Download Figure 6-1, TIF file.

10.1523/JNEUROSCI.0112-22.2023.f6-2 Extended Data Figure 6-2 Individual speech-induced oscillatory power in the ASD group. Download Figure 6-2, TIF file.

Although results from mid-central electrodes showed significant PAC clusters between the 3- to 8-Hz (phase) and the 22- to 38-Hz (amplitude) frequency ranges in both groups, there was only minimal overlap between ASD and TD (Fig. 6*B*). While TD showed a unique and strong δ-θ/low-γ PAC on mid-central (Fig. 6*B*) and posterior-occipital electrodes (Fig. 6*D*), very young children with ASD exhibited wholly different patterns. The most significant difference was the extra presence of a consistent low-β/low-γ PAC in both mid-central and posterior-occipital electrodes (Fig. 6*D*). In summary, compared with the TD group, children with ASD show atypical δ/θ-γ coordination, and a robust low-β/low-γ coupling anomaly.

### Prediction of clinical variables from oscillatory neural features

Among all the oscillatory features that were found atypical in children with ASD, not all of them have the potential to specifically account for individual traits and, in particular, language abilities. We, therefore, assessed whether the group differences observed at the neurophysiological level could first predict the ASD severity, and, more importantly, whether they could predict the speech reception scores obtained by the children in the MSEL (direct assessment of developmental functioning) and VABS-II (parent-reported measure of functioning in everyday life). Conversely, we also sought to find out whether some of the oscillatory anomalies detected in power, tracking, and PAC analyses, could be more generally involved in several other cognitive skills (verbal production, visual processing, and fine motor skills).

### Predicting ASD severity (ADOS severity level)

**Figure 7.**
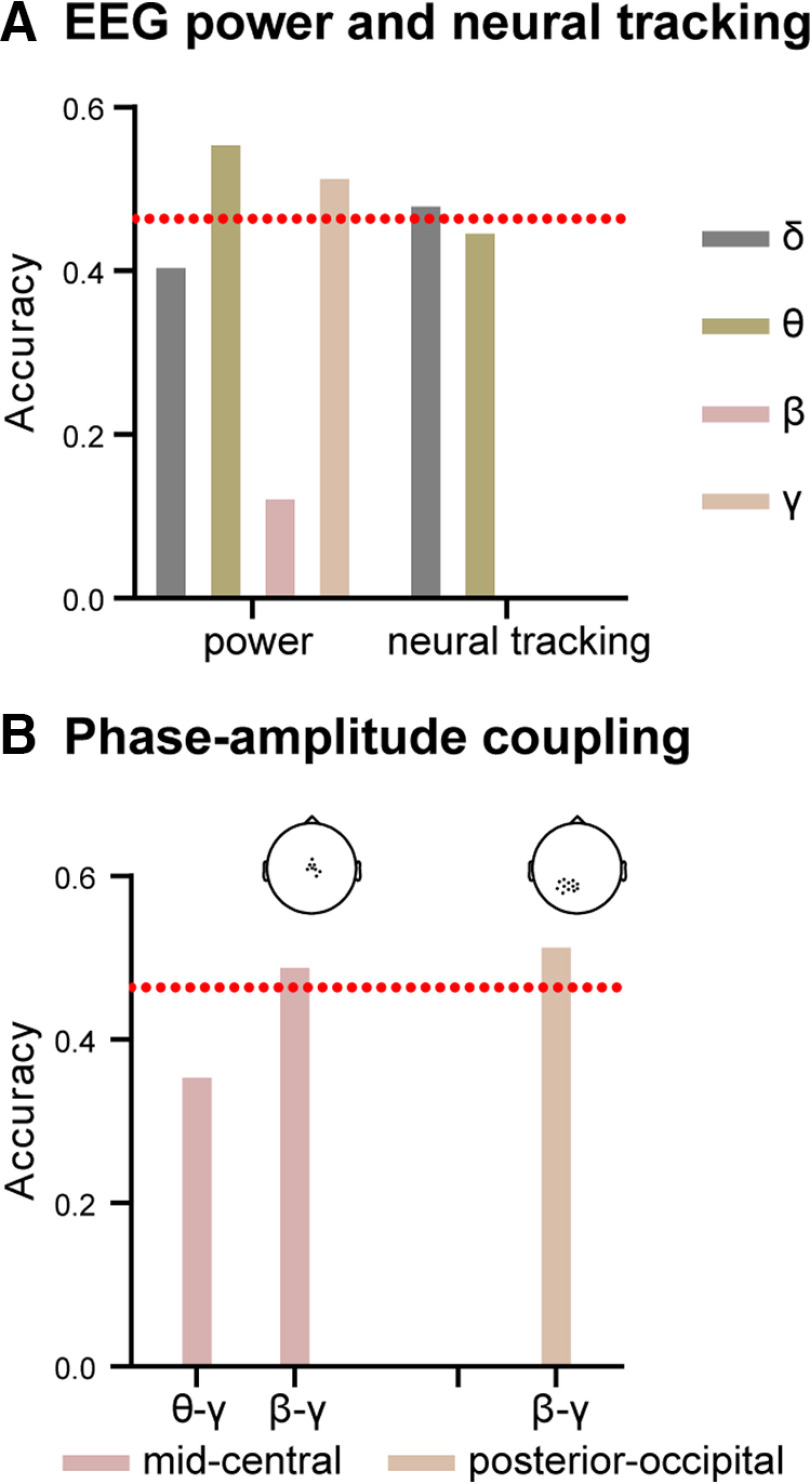
Predicting ASD severity from EEG oscillatory activity. ***A***, Prediction accuracy of ASD symptom severity using the EEG power, neural tracking based on all electrodes. ***B***, Prediction accuracy of ASD symptom severity using the phase-amplitude coupling (cluster-based). The red dash line shows the chance level determined by an inverse binomial distribution. For the exact location of clusters, see scalp topographies on top of panel ***B***.

We first used nested LDA classification to determine which neurophysiological feature(s), i.e., EEG power, speech-tracking values, and PAC, best predicted the ASD symptom severity level, i.e., low, moderate, and high. Classification using all scalp electrodes showed that θ power (accuracy: 55.8%), low-γ power (accuracy: 51.7%), δ tracking (accuracy: 48.3%) were good predictors of autism severity (empirical chance level: 46.387%; Fig. 7*A*).

In addition, low-β/low-γ PAC was a good predictor of autism severity in both clusters [accuracy 49.2% in the mid-central cluster and 51.7% in the posterior-occipital cluster (Fig. 7*B*), empirical chance level: 46.387%]. These results demonstrate that θ power, γ power, δ tracking, β/γ coupling contain critical information about autism severity.

### Predicting speech reception level

**Figure 8.**
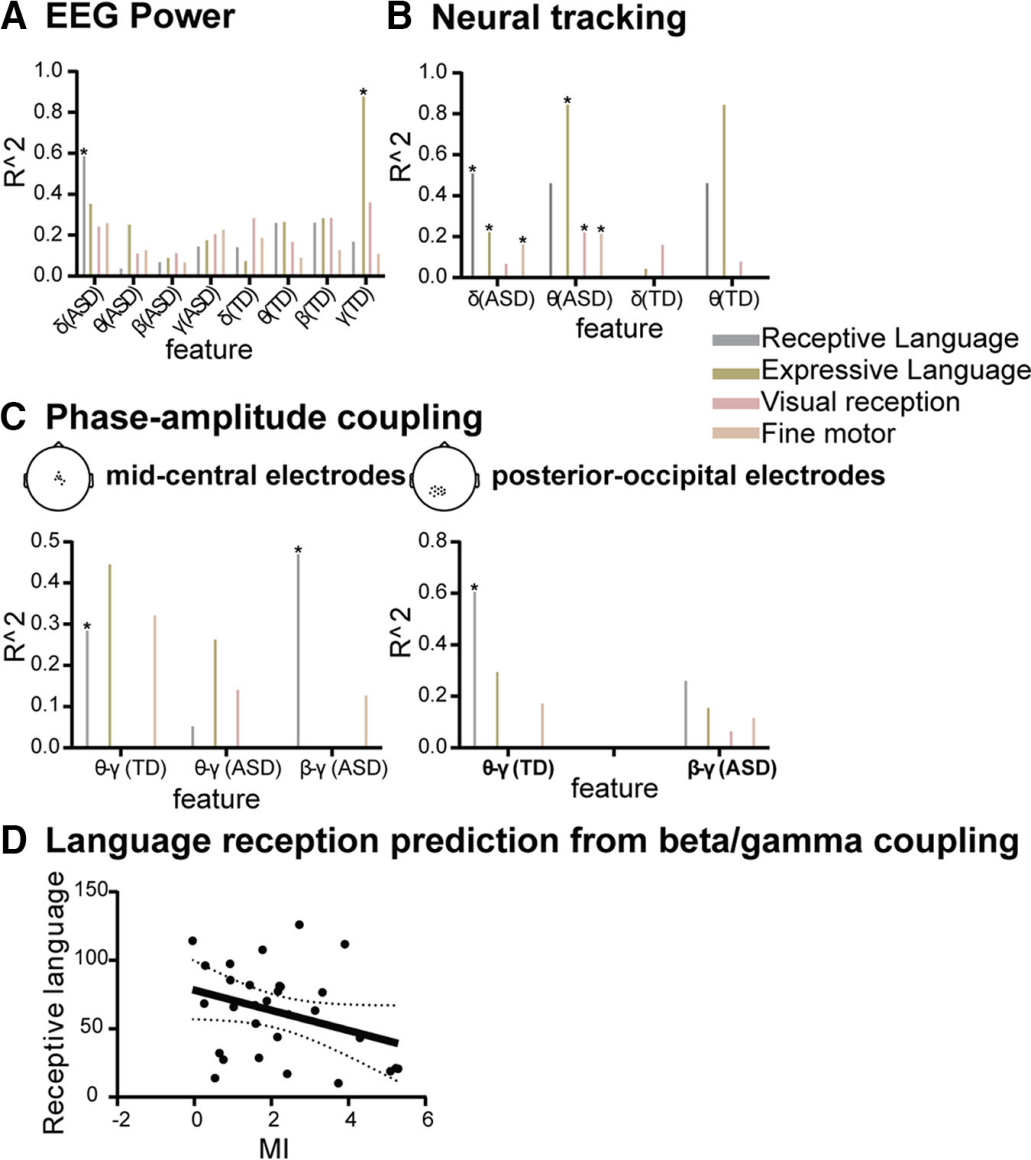
Predicted development levels in young children from EEG oscillatory activity using a regularized linear model (Lasso). ***A***, ***B***, Low-γ power significantly predicts Language expression in TD (***A***), δ-/θ-tracking significantly predicts all tested cognitive components but language reception for θ tracking in ASD, and none in TD (***B***). ***C***, θ/low-γ PAC specifically predicted language reception in TD (***C***), whereas β/low-γ specifically predicted language reception among young children with ASD (***C***, left panel). *R*^2^ values represent the proportion of the variance that is explained by the features for each target variable. ***D***, Language reception prediction from β-γ coupling (*r* = −0.33, *p* = 0.04). Asterisks indicate the significant *R*^2^, *p* < 0.05; δ refers to δ, θ refers to θ, β refers to low-β, γ refers to low-γ. For the exact locations of clusters, see the scalp topographies on top of each panel in ***C***. The missing bars indicate the *R*^2^ is close to zero.

**Figure 9.**
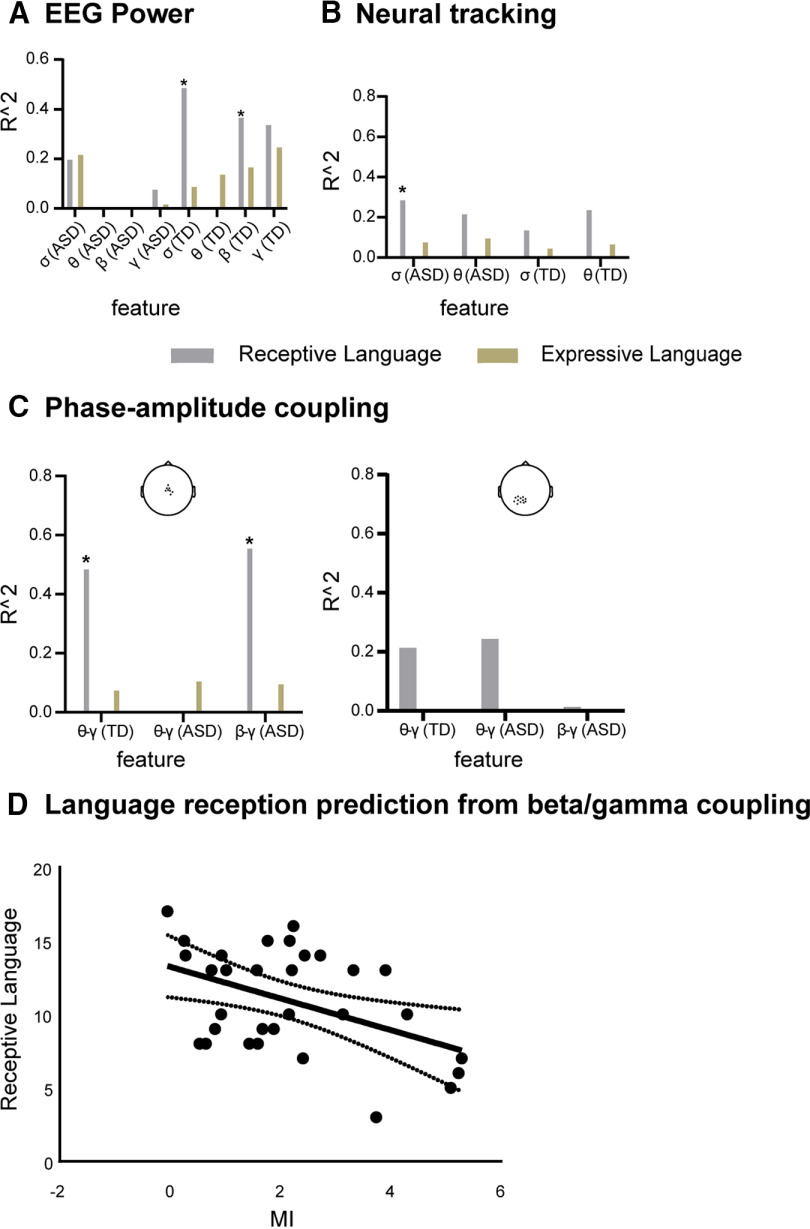
Predicted VABS-II (Vineland Adaptive Behavior Scales) development level in young children from EEG oscillatory activity using a regularized linear model (Lasso). ***A***, ***B***, δ And β power significantly predict language reception in TD (***A***), and δ tracking significantly predicts language reception in ASD (***B***). ***C***, θ/low-γ PAC specifically predicts language reception in TD (***C***), whereas β/low-γ specifically predicts language reception among young children with ASD (***C***, left panel). *R*^2^ values represent the part of the variance that is explained by the features for each target variable. ***D***, Language reception prediction from β-γ coupling (*r* = −0.45, *p* = 0.01). Asterisks indicate significant *R*^2^, *p* < 0.05; δ refers to δ, θ refers to θ, β refers to low-β, γ refers to low-γ. For the cluster locations, see corresponding scalp topographies on each panel ***C***. The missing bars indicate the *R*^2^ is close to zero.

We then used Lasso, with a nested cross-validation approach, to determine whether individual language skills could be predicted from oscillatory features and which features accounted most selectively for individual language development status assessed by MSEL (Fig. 8) and VABS-II (Fig. 9). The analyses were run separately in each group. Although different in TD and ASD groups, band-specific EEG power accounted for none of the cognitive subscales of MSEL in any groups, except expressive language in TD children for the γ power and receptive language in children with ASD for the δ power (Fig. 8*A*). Conversely, neural tracking, which was also markedly different across groups, was predictive of three cognitive subscales of MSEL in ASD (receptive and expressive language and fine motor skills) in the δ range, and of all but receptive language in the θ range (Fig. 8*B*). In the TD group, neural tracking accounted for none of the MSEL scores, despite a trend for θ tracking to predict both language reception and expression. These results were also found for the language components as assessed by VABS-II (Fig. 9*B*). In TD, the *R*^2^ values were very low for all cognitive aspects in the δ range, and for motor and visual in the θ range (Fig. 8*B*).

**Figure 10.**
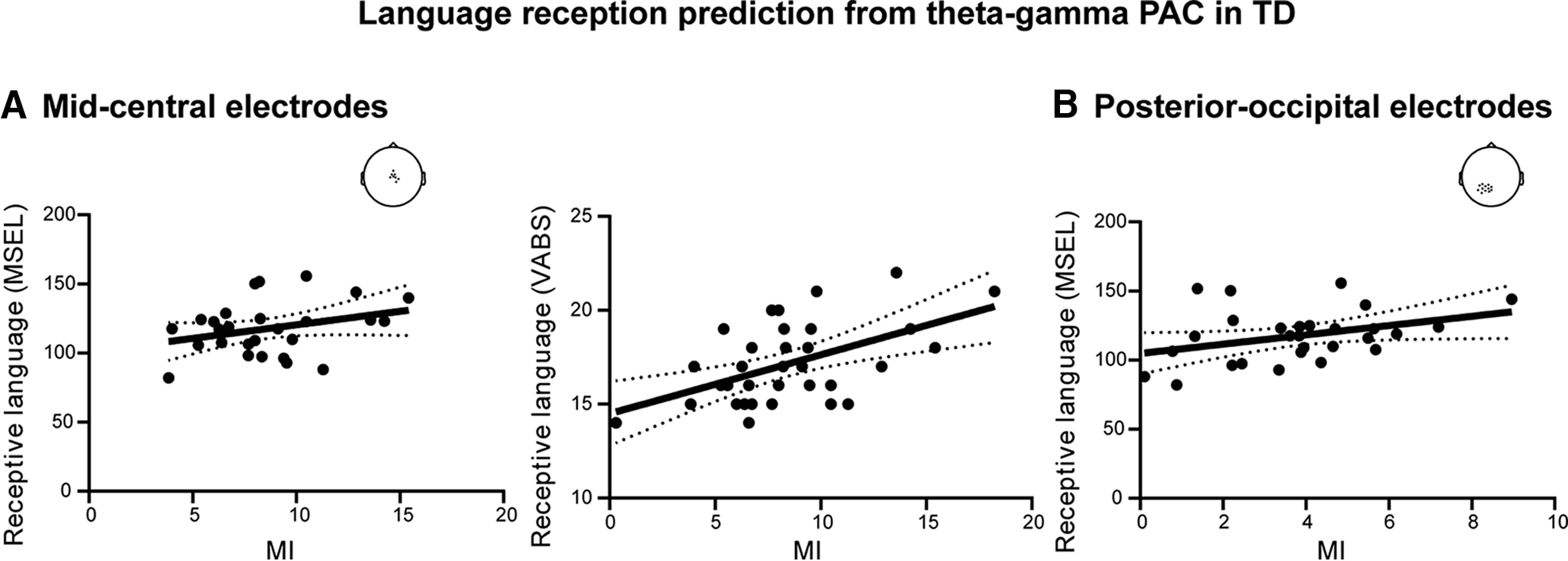
Language reception prediction from θ-γ coupling (PAC) in TD. ***A***, Predicted language reception from cross-frequency coupling (MI) sampled from mid-central electrodes (r_MSEL_ = 0.30, *p* = 0.05; r_VABS_ = 0.52, *p* = 0.002). ***B***, Predicted language reception from cross-frequency coupling (MI) sampled from posterior-occipital electrodes (*r* = 0.36, *p* = 0.03). For exact cluster locations, see scalp topographies.

The most relevant feature for predicting language reception development was phase-amplitude coupling. As expected from studies in typical adults (Gross et al., 2013; Lizarazu et al., 2019) and from neurocomputational models (Hyafil et al., 2015a,b), θ/γ coupling selectively explained language reception in both central and posterior-occipital clusters of electrodes in our group of very young TD children (Figs. 8*C*, 9*C*, 10). In the ASD group, the (atypical) β/γ PAC selectively predicted language reception (Fig. 8*C*, 9*C*). The *post hoc* analysis of the dependency of language reception on β/γ PAC indicated that the stronger the anomaly, the worse the speech reception was (Figs. 8*D*, 9*D*). For display purposes, after confirming the performance of the algorithm, we used whole data to run the hyperparameter optimization, and with the best estimator we then retrained the algorithm y = f(x). We, therefore, obtained a fitted model y = 17.45·*f*_p_ + 0.43·*f*_a_-5.71·MI-142.19 (for MSEL) and y = 0·*f*_p_ + 0·*f*_a_-0.62·MI + 12.26 (for VABS-II), in which y represents language reception, *f*_p_ and *f*_a_ represent the frequency of phase and amplitude respectively, and MI refers to PAC value. Overall, PAC was the most specific predictor of language reception in ASD and TD children: the presence of θ/γ PAC predicts good speech reception in TD children, whereas the added presence of atypical β/γ PAC signals poor reception in ASD. Importantly, PAC features were much more sensitive than power and neural tracking to predict individual language reception scores.

## Discussion

The goal of this study was to determine whether speech-related oscillatory anomalies in ASD are already present in early childhood, around the time of ASD diagnosis. Given the established computational role of neural oscillations in chunking the syllable stream, encoding phonemic information, and predicting speech timing and linguistic content (Giraud and Poeppel, 2012; Hovsepyan et al., 2020), we also sought to determine which of several potential anomalies could most specifically be associated with speech reception difficulties in ASD. Establishing the specific relevance of oscillation anomalies with respect to speech development is critical, as early targeted neural interventions could subsequently be envisaged to normalize speech reception, as recently demonstrated in other neurodevelopmental language disorders (Ladanyi et al., 2020; Marchesotti et al., 2020). Exploring EEG in 64 children between 1.31 and 5.56 years old with balancing the gaze-divergence across groups, we found marked anomalies of speech-induced cortical activity in the group with ASD, including decreased expression of δ, low-γ, and β frequencies. While θ power appeared as pronounced among ASD and TD children, δ-θ neural tracking was significantly reduced in ASD. Our most important results were observed in relation to oscillation cross-frequency coupling, which reflects the coordination computations at different timescales. As expected from previous studies in adults (Gross et al., 2013; Hyafil et al., 2015a; Jochaut et al., 2015; Lizarazu et al., 2019), we clearly detected the classical θ/γ coupling in very young (approximately three years old) TD children and found this feature to predict their individual language reception scores specifically. This result represents per se an important finding supporting the notion that PAC is not a simple marker of speech reception ability, but reflects a key computational component of speech processing, namely the hierarchical relationship between phonemes and syllables. This typical θ/low-γ PAC was altered in children with ASD, appearing over a shifted θ/γ range and lacking occipital location once the visual exploration contribution across groups was controlled for. Critically, θ/γ coupling was accompanied by a nontypical low-β/low-γ PAC in the ASD group, which, among all the abnormal oscillatory features reported here, was the only one from which we could specifically predict language reception scores in children with ASD: the stronger the β/low-γ coupling, the worse the speech reception. This key finding suggests that the speech processing computational scales are markedly different in ASD. We acknowledge, however, that because of larger interindividual variability in the neural sources among ASD children compared with TD controls, group-level statistics may appear weaker in ASD (Hasson et al., 2009). Source space analyses, which in our case were not possible because of the absence of structural MRI data and real digitized head points, could have led to slightly different observations. The cartoon video contained acoustic and visual features, triggering audiovisual integration. Given that modality preference can influence performance on multimodal tasks (Sumby and Pollack, 1954; Calvert et al., 1997; McDonald et al., 2000; Slutsky and Recanzone, 2001; Morein-Zamir et al., 2003; Feng et al., 2017; Lee et al., 2018; Robinson and Sloutsky, 2019), we cannot exclude that atypical speech perception in children with ASD is influenced by how individuals with ASD process visual information. Although our statistical analysis controlled for gaze patterns, differences between typical and ASD children with respect to cross-modal interactions are likely, and future research should investigate how visual information interacts with auditory neural entrainment in children with and without ASD.

### Low-γ power predicts language expression in typically developing children

Our results showed decreased low-γ activity among ASD relative to TD children, notably on mid-central electrodes, a scalp location that strongly captures auditory cortex activity (Stropahl et al., 2018). Previous studies already reported reduced γ activity in ASD in response to pure tones (Rojas et al., 2008; Gandal et al., 2010), presumably denoting a basic functional anomaly of the auditory cortex. γ activity usually reflects the excitation-inhibition balance (Buzsaki and Wang, 2012) within brain circuits, which is a core parameter in neural development. Reduced low-γ power in ASD could be seen as a probe of atypical maturation of auditory neural circuitry. At the computational level, low-γ activity is associated with phonemic encoding (Lehongre et al., 2011; Hyafil et al., 2015a) and its nesting within θ rhythm is associated with the encoding of phonemic information within syllabic frames, enabling syllable-level representations to interface at the right time with other (higher) processing stages (Ghitza, 2011). Here, we found that low-γ activity was the only feature that specifically predicted language expression among TD children, a sensible finding as language expression is tightly related to the transformation of phonetics into articulatory features at the same timescale (Chartier et al., 2018).

### Abnormal θ-range speech tracking and θ/γ coordination

Although globally we found a similar level of θ activity in both groups, θ-range tracking was deficient among children with ASD, meaning that θ activity, although present, did not typically follow the speech temporal structure. An equivalent level of global θ power in TD and ASD is consistent with previous studies (Wang et al., 2017; Zhang et al., 2018). However, the fact that the speech envelope was less accurately reconstructed from the EEG signals among ASD compared with TD children indicates that θ activity in ASD is more weakly engaged in syllable tracking (Giraud and Poeppel, 2012; Hyafil et al., 2015a; Pefkou et al., 2017). This anomaly likely disrupts the alignment of neuronal excitability with syllabic onset, weakens the coordination of speech with oscillations on other frequency bands, notably the low-γ one, and ultimately hampers phonemic information encoding within syllables (Lehongre et al., 2011; Gross et al., 2013; Hyafil et al., 2015a; Lizarazu et al., 2019; Hovsepyan et al., 2020). Accordingly, we found anomalies of the classical speech-specific θ/γ PAC among children with ASD. This anomaly was not explained by differences between groups in the way children screened the visual scene, as gaze differences were controlled for. The coupling was shifted toward lower γ frequencies, making it incompatible with a typical role in phonemic sampling within left auditory regions, as reported in healthy individuals (Poeppel, 2003; Morillon et al., 2010). These results, however, are only in partial agreement with the more severe anomalies of θ/γ coupling, a fully inverted coupling relationship, that were previously observed using simultaneous fMRI-EEG among adults with ASD (Jochaut et al., 2015). Longitudinal studies are needed to determine whether θ/γ coupling further deteriorates during childhood development and adolescence.

### Reduced δ-power but not δ-range speech tracking is a specific predictor of language reception in ASD

Our results also show reduced δ power and δ-range speech tracking among children with ASD. δ-range activity signals phrase-level chunks, which do not necessarily have a physical/acoustic counterpart in the speech stimulus (Boucher et al., 2019). δ Activity is known to reflect more endogenous processes than θ-range syllable tracking (Molinaro and Lizarazu, 2018), which are argued to pertain to syntactic grouping (Ding et al., 2014; Meyer et al., 2017) or prosody processing (Ghitza, 2017; Teoh et al., 2019). The fact that reduced δ activity predicted speech reception in ASD might therefore denote altered syntactic phrasal chunking and is compatible with previous observations suggesting both weaker linguistic (Haesen et al., 2011) and intonation processing (Benitez-Burraco and Murphy, 2016). Although our results align well with these previous observations and hypotheses, δ-range speech tracking did not only predict speech reception scores but also expressive language, and fine motor skills (assessed with MSEL), suggesting the role of these oscillatory features in ASD goes beyond the sole domain of speech reception. Despite the collinearity across MSEL subscales, neural tracking also predicted language development when assessed with VABS-II. While θ tracking characterized language development in TDs, individuals with ASD showed δ tracking instead. Logically, the more endogenous δ tracking deficit has a more global impact on the cognitive profile of children with ASD and could also be a parameter that could possibly be adjusted using adapted neurostimulation methods.

### β/γ Cross-frequency coupling: a speech reception singularity in young children with ASD

During continuous speech perception, top-down predictive mechanisms are also important, particularly to make sense of acoustic signals that might be unclear or ambiguous, or simply to follow the speaker’s speech rate. Typically, predictive mechanisms are signaled by the low-β band (Fontolan et al., 2014), which we found to be weaker among ASD compared with TD children around the mid-central region. Low-β neural activity is argued to mediate top-down information passing (Chao et al., 2018; Bastos et al., 2020), allowing for precise temporal (Fujioka et al., 2012) but also content-specific predictions (Chao et al., 2018). In speech processing, this frequency band could provide top-down integration constants that are intermediate between θ-syllabic and γ-phonemic ranges (Pefkou et al., 2017; Giraud, 2020). By alternating with bottom-up γ phases (Fontolan et al., 2014), β activity phases could stabilize representations in the face of the ever-changing acoustic input. Therefore, weaker β activity in ASD might reflect a reduced deployment of predictive mechanisms, altering both the ability to predict when acoustic signals can be expected and what content they carry. This interpretation is globally in line with alterations of phasic predictive learning in a mouse model of autism (Kosaki and Watanabe, 2016) and more generally with the hypothesis of impaired predictive coding in ASD (Courchesne and Allen, 1997; Sinha et al., 2014; Van de Cruys et al., 2014).

The key finding of this report is the atypical low-β/low-γ PAC found among children with ASD in conjunction with weaker β power. A PAC anomaly involving the low-β phase might follow from the reduced global β power in ASD, possibly allowing low-γ and γ bursts to occur more strongly within β troughs. Reduced β gating could result in letting past neural activity that is unrelated to the predicted speech structure, possibly leading to a feeling of being overwhelmed by unformatted acoustic inputs to which no linguistic value or meaning can be attributed. The presence of an abnormal low-β/low-γ coupling in ASD was not influenced by atypical visual scene exploration in ASD, as possible gaze differences were controlled for in the statistical analysis. This interpretation aligns well with the clinically described auditory avoidance in ASD children (Haesen et al., 2011; Top et al., 2018). The other hypothesis would be the existence of a stronger β state where new sensory input is less easily incorporated, and where subjects are in a dominant “internally driven state,” similar to schizophrenic symptoms (Grace, 1991). According to the communication-through-coherence theory (Fries, 2005, 2015), this remarkable β-γ PAC anomaly could be associated with the persistence of the status quo state and thus less flexibility in cognitive control (Engel and Fries, 2010; Herring et al., 2019; Wagner et al., 2019). The low-β/low-γ coupling may suggest a greater endogenous/exogenous processing ratio in ASD, particularly when exposed to speech stimuli. The observed abnormalities in neural speech processing could result from strong endogenous neural patterns, leaving subjects locked into existing “internally driven states” with a reduced capacity to shift to and integrate novel sensory information (Pogosyan et al., 2009). The difficulty in processing new and fast-changing sensory stimuli is likely associated with a reduced amount of down-propagating prediction errors.

### Cross-frequency oscillation features: a potential endophenotype for targeted interventions

As cortical oscillations arise from excitatory-inhibitory interactions within and across specific cortical laminae (Cannon et al., 2014), auditory oscillation anomalies represent a plausible functional counterpart to structural disorganization and disruption of cortical inhibition previously shown in ASD (Rojas et al., 2008; Gandal et al., 2010). A recent study directly relates neural oscillations with the expression of numerous genes, several of which are involved in ASD (Berto et al., 2021), e.g., LNX1, DGKI, KCNQ5, DCX, SHANK2, etc. Therefore, speech reception difficulties in ASD could directly result from structural anomalies induced by mutations of genes (Caubit et al., 2016) controlling neuronal interactions, notably at the synaptic level (Caubit et al., 2016).

Disruptions of synchronous neural activity in the cortex (Dinstein et al., 2011) and other brain structures, such as the cerebellum and hippocampus (Donovan and Basson, 2017), could lead to fragmented speech processing, abstraction difficulties in the auditory modality, and difficulties to map atypical auditory representations into appropriately timed articulatory sequences. Here, we found that among many neural oscillation anomalies, the most promising features lie in cross-frequency coupling patterns, particularly the θ/γ coupling that has an abnormal topography in ASD and the low-β/low-γ coupling that is wholly atypical. These two anomalies could be an ideal entry point for targeted brain stimulation interventions aiming at downregulating the abnormal low-β/low-γ coupling, re-localizing the typical auditory θ/low-γ coupling to left auditory regions, and upregulating θ/γ coupling that is classically observed in the superior temporal cortex region. The next step in this line of research will be to test whether a simple θ/γ stimulation at the exact right frequencies (5 Hz by 30 Hz) using, e.g., mild transcranial alternating current stimulation could indeed both disrupt low-β/low-γ and relocate θ/low-γ activity to auditory regions. In combination with close monitoring of behavior and EEG activity, such a trial would also allow us to firmly establish a direct link between auditory oscillatory activity and the speech reception ability in children with ASD.

In conclusion, our results confirmed the relevance of θ/low-γ coupling in speech reception in very young TD children and showed that cross-frequency patterns were markedly disrupted in children diagnosed with ASD. While θ/γ coupling closely predicted individual language reception abilities in TD children, the speech reception deficit in ASD was predicted by the unusual presence of β/γ coupling in auditory-sensitive brain regions. Cross-frequency coupling features appear as a promising language development endophenotype, bridging the gap from genetics to behavior while offering a precise entry point for interventions targeting the normalization of cross-frequency oscillatory functions. Although our study alone cannot establish causality, demonstrating the presence of oscillatory anomalies at the age of diagnosis is an important step toward reaching a causal explanation for speech reception difficulties in ASD.
