# Supplementary figures and images for: Speech Reception in Young Children with Autism Is Selectively Indexed by a Neural Oscillation Coupling Anomaly
Source: J Neurosci. 2023 Oct 4;43(40):6779–95. doi: 10.1523/JNEUROSCI.0112-22.2023 (PMC10552944; doi:10.1523/JNEUROSCI.0112-22.2023)

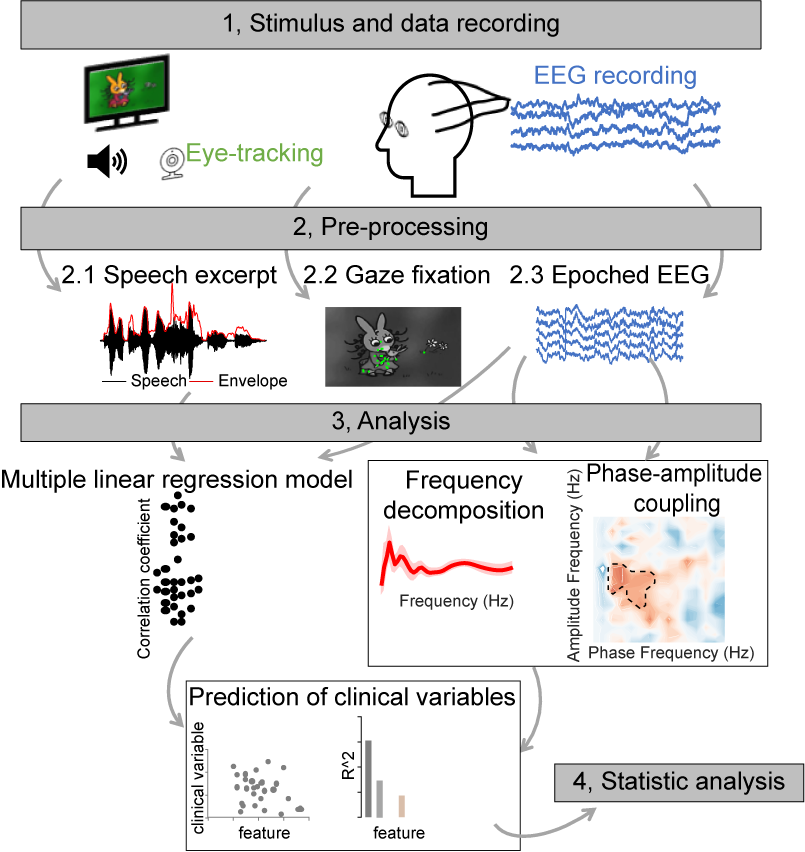

Supplement: Extended Data Figure 1-1 — Stimulus properties and schematic of the processing pipeline. There were four main steps of the processing: stimulus presentation and EEG and eye-tracking data recording (step 1), preprocessing of stimuli (step 2.1, extracting speech envelope, steps 2.2 and 2.3 eye-tracking and EEG data preprocessing), data processing based on purpose (step 3 left up, neural tracking via multiple linear regression model; step 3 right up, frequency decomposition via wavelet transformation and phase-amplitude coupling via KL-MI-Tort approach; step 3 bottom, prediction of clinical variables via LASSO); and last step, step 4, between-group statistical analysis. For detailed information on each step, please see Materials and Methods. Download Figure 1-1, TIF file. [file ns-JN-RM-0112-22-s01.tif]

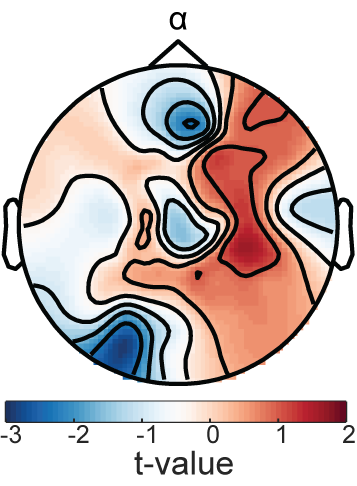

Supplement: Extended Data Figure 4-1 — Comparison of α frequency power in 31 children with ASD and 33 TD peers. Children with ASD had comparable α power relative to their TD peers (cluster-based nonparameters permutation tests, cluster corrected p = 0.05). Download Figure 4-1, TIF file. [file ns-JN-RM-0112-22-s02.tif]

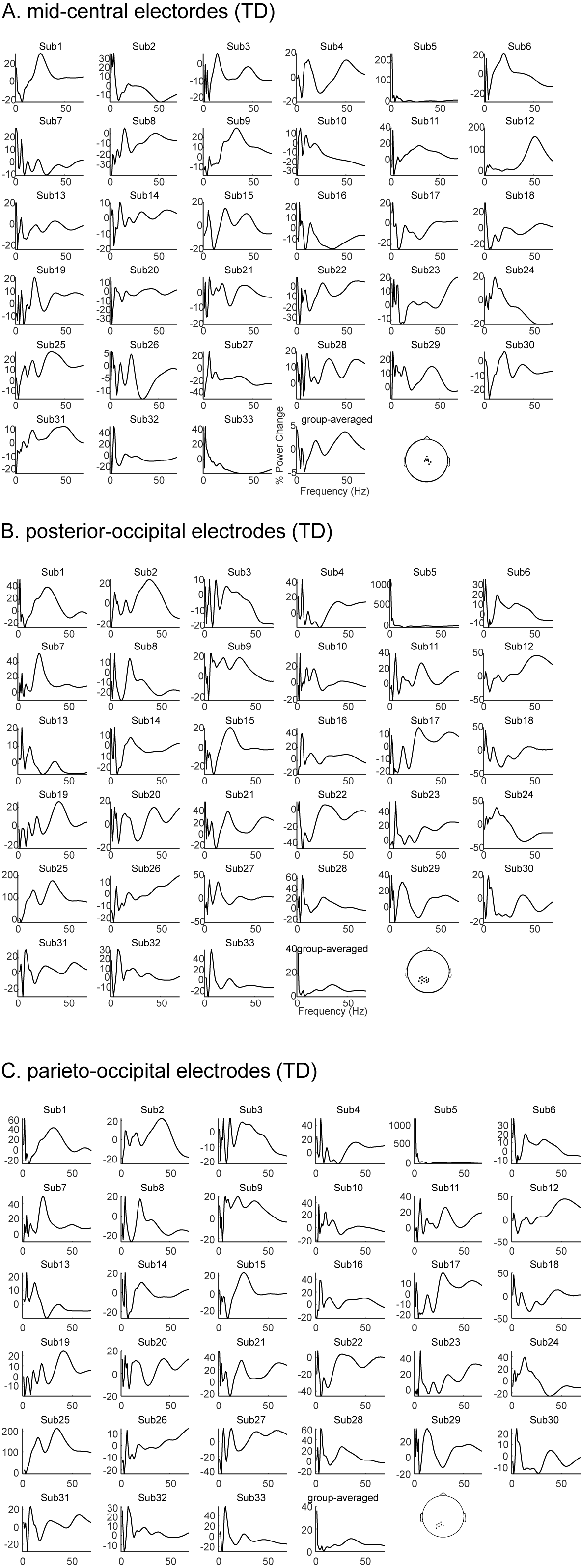

Supplement: Extended Data Figure 6-1 — Individual speech-induced oscillatory power in the TD group. Download Figure 6-1, TIF file. [file ns-JN-RM-0112-22-s03.tif]

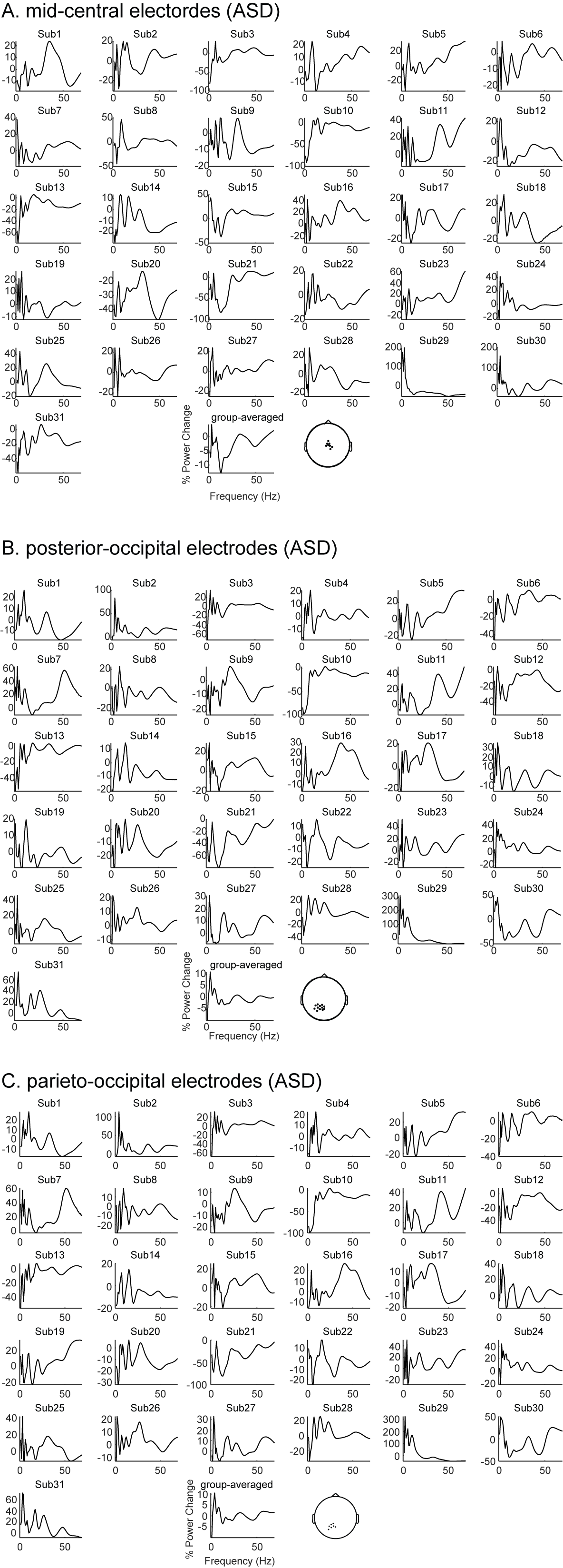

Supplement: Extended Data Figure 6-2 — Individual speech-induced oscillatory power in the ASD group. Download Figure 6-2, TIF file. [file ns-JN-RM-0112-22-s04.tif]
